# Modelling Temperature-dependent Schistosomiasis Dynamics for Single and Co-infections with *S. mansoni* and *S. haematobium*

**DOI:** 10.1371/journal.pone.0318720

**Published:** 2025-03-13

**Authors:** Zadoki Tabo, Lutz Breuer, Christian Albrecht

**Affiliations:** 1 Department of Animal Ecology and Systematics, Justus Liebig University Giessen, Giessen, Germany; 2 Department of Landscape Ecology and Resource Management, Justus Liebig University Giessen, Giessen, Germany; 3 Centre for International Development and Environmental Research (ZEU), Justus Liebig University Giessen, Giessen, Germany; Niigata University of Pharmacy and Medical and Life Sciences, JAPAN

## Abstract

Schistosomiasis, a prevalent public health issue specifically in sub-Saharan Africa, is primarily attributed to *Schistosoma haematobium* and *Schistosoma mansoni*, often occurring concurrently. These schistosome species share similarities in life cycles and transmission, manifesting comparable infection patterns and susceptibility to temperature variations. This study investigates the influence of temperature control not only on the transmission of individual species but also on their mutual interactions and co-infection dynamics using a mathematical model. Sub-models and co-dynamic properties, including reproduction numbers, equilibrium states, and stability conditions, are derived. Sensitivity analysis is performed to clarify the impact of parameter variations on model stability. Results suggest that temperature variation increases the spread of *S. haematobium*, which enhances susceptibility to *S. mansoni* co-infection, possibly by altering the immune response. At moderate temperatures (20°C and 25°C), infection levels in both single and co-infected individuals are higher, while recovery rates increase with temperature, peaking at 25°C and 35°C as infections significantly decrease. *Biomphalaria* snails exhibit greater population growth and susceptibility to infection than *Bulinus* snails, particularly below 25°C. Above this temperature, *Biomphalaria* population decreases while *Bulinus* species are more likely to experience faster mortality. These temperature-related variations differently impact mortality rates of intermediate snails and snail-to-human transmissibility rates for schistosome species, holding significant health implications. Targeting snails during seasons below 25°C, when susceptibility is higher, and intensifying human treatment interventions around 25°C–35°C, where recovery rates peak, may yield optimal results, particularly during seasons with intermediate temperatures around 25°C for both snails and humans. The results underscore the importance of integrating temperature into models for predicting and managing schistosomiasis dynamics for both genera. Therefore, this model is applicable not only to sub-Saharan Africa, but also to other regions where the described temperature ranges match with the local climate.

## 1. Introduction

Schistosomiasis, a neglected tropical disease (NTD), is widely prevalent in sub-Saharan Africa characterized by poverty and limited access to safe drinking water and proper sanitation facilities [1]. The disease poses a significant health risk to the population, with annual death rates estimated to be around 200,000 to 280,000 deaths per year in the region [2]. It is important to note that these figures are subject to change as new data becomes available as efforts to control and treat schistosomiasis continue to progress [1]. Schistosomiasis is caused by parasitic worms known as *Schistosoma* trematodes, or blood flukes. These worms are transmitted to humans through intermediate host snails [3,4]. While various species of schistosomes can infect humans [5], *Schistosoma mansoni* and *Schistosoma haematobium* are particularly prevalent and exert a significant burden on countries in sub-Saharan Africa [4]. These two types of schistosomes are closely related and have similar complex life cycles and transmission dynamics, but they differ in their distinct pathological profiles. *Schistosoma mansoni* causes intestinal schistosomiasis and is transmitted by all snail species of the *Biomphalaria* genus within the Planorbidae family [6]. On the other hand, *S. haematobium* causes urogenital schistosomiasis and is transmitted by specific snails belonging to the *Bulinus* genus within the Bulinidae family [7].

The life cycle of the schistosome begins with cercaria shedding from infected snails into the water. Within a few seconds of contact with the human host, they can penetrate through the skin and invade the body. The cercaria larvae enter the circulatory system and migrate through the lungs to the liver where they transform into adult schistosomes and mate inside the body. Subsequently, adult couples migrate to their final destination to reproduce. *Schistosoma mansoni* moves to the blood arteries and the portal system where the females release their eggs through the intestinal walls and are expelled from the body in feces into freshwater sources. Conversely, *S. haematobium* migrates to the vessels of the urinary bladder, where females produce eggs that pass through the bladder wall and are excreted in the urine contaminating freshwater sources. The eggs of both schistosome species hatch into miracidia larvae in freshwater. These larvae exclusively infect respective intermediate host snails, where they undergo transformation into cercaria larvae. Subsequently, these cercariae infect humans, completing the life cycle [8,9].

Human schistosomiasis has a wide-ranging impact on various organ systems, affecting the cardiopulmonary, gastrointestinal, genital, and central nervous systems. Infections caused by *S. mansoni* may result in complications such as pulmonary hypertension and schistosomal appendicitis, and many other [1,10,11]. In contrast, *S. haematobium* infection manifests with symptoms including, but not limited to, hematuria, bladder cancer, anemia, and infertility [1,12]. Additionally, co-infection with both *S. haematobium* and *S. mansoni* can lead to a more complicated immune-mediated glomerulopathies [13]. Currently, the anthelmintic praziquantel remains the primary treatment for both schistosomiasis forms due to its efficacy against adult worms, while no effective vaccine is yet available [14]. Although praziquantel reduces worm burden, it may not eliminate immature worms or eggs in the body, and concerns persist regarding drug resistance and re-infections after treatment [15]. To manage schistosomiasis effectively, continued research and surveillance are essential [16]. Hence, accounting for the specific species (genera) involved in both single infections and mixed co-infections of schistosomiasis is essential for disease treatment, control, and overall human health as humans functions as the definitive host.

In sub-Saharan Africa, persistent transmission of both single and mixed schistosomiasis can be influenced by various factors, including climate change and global warming. Among these factors, temperature plays a significant role in snail distribution and population size, affecting some traits of the schistosome life cycle and overall human infection dynamics [17,18]. The differing responses of hosts and parasites to temperature fluctuations can either elevate or reduce disease prevalence [19]. While literature links schistosomiasis to various diseases [20–22], existing mathematical models focus on co-infections but lack consideration of climate change. These models explore schistosomiasis interactions with other diseases but overlook *Schistosoma* species-to-species interaction and climate change factors [23–27]. Furthermore, laboratory experiments conducted by Mangal et al. [17] on the *Biomphalaria-S. mansoni* system and Kalinda et al. [18] on the *Bulinus-S. haematobium* system have yielded crucial temperature-dependent data regarding schistosome life cycle traits. Utilizing models parameterized by such data becomes imperative, especially in regions like Sub-Saharan Africa, particularly East Africa, where local temperatures correspond to temperature ranges observed in these experiments and where both infections coexist [2,22]. Despite the significance of this data, a noticeable gap exists in the absence of a mathematical model that employs this information to elucidate the impact of temperature variations on schistosomiasis transmission, distinguishing between single-species infections and their mixed co-infection interactions. This study aims to develop a globally applicable mathematical model, inspired by the classic SIR model, to quantitatively predict temperature control over the trends, interactions, and differences in *S. mansoni* and *S. haematobium* co-infection dynamics for effective disease control planning. The selection of the SIR model for investigating the spread of single and mixed schistosomiasis infections at the population level agrees with similar co-dynamic models found in the existing literature [25–27]. However, our study uniquely incorporates temperature as a crucial factor influencing transmission rates, *Schistosoma* species interaction, and co-infection dynamics, which provides new insights into the optimisation of intervention strategies based on climatic variations.

## 2. Materials and methods

### 2.1. Co-dynamics model formulation and equations

In our co-dynamics model studying the interactions between *S. mansoni* and *S. haematobium*, we divide the total human population ($N_{h}$) into different subpopulations. These subpopulations include susceptible humans (H), individuals infected with only *S. mansoni* ($I_{m}$), individuals infected with only *S. haematobium* ($I_{h}$), individuals infected with both strains ($I_{hm}$), individuals who have recovered from *S. mansoni* ($R_{m}$), individuals who have recovered from S*. haematobium* ($R_{h}$), and individuals who have recovered from both strains ($R_{hm}$). Similarly, the total population of *Biomphalaria* snails ($N_{1}$) is divided into susceptible snails ($S_{1}$) and infected snails ($I_{1}$), while the total population of *Bulinus* snails $(N_{2}$) is divided into susceptible snails ($S_{2}$) and infected snails ($I_{2}$). Mathematically, we can express these subdivisions as follows: $N_{h}=H+I_{m}+I_{h}++I_{hm}+R_{m}+R_{h}+R_{hm}$_,_ $N_{1}=S_{1}+I_{1}$*,* and $N_{2}=S_{2}+I_{2}$. Consequently, we describe a co-dynamics model using a system of ordinary differential equations Eqs. (1)–(11).


$$H^{\prime }=\Lambda_{h}+\varepsilon R_{h}+\alpha R_{m}+\theta R_{hm}-\left(\beta_{i}\left(T\right)I_{2}+\beta_{u}\left(T\right)I_{1}\right)H-\upsilon_{1}H$$ (1)

$$I_{m}^{'}=\beta_{i}\left(T\right)I_{2}H-\beta_{u}\left(T\right)I_{1}I_{m}-\left(\gamma +\delta_{i}+\upsilon_{1}\right)I_{m}$$ (2)

$$I_{h}^{'}=\beta_{u}\left(T\right)I_{1}H-\beta_{i}\left(T\right)I_{2}I_{h}-\left(\omega +\delta_{u}+\upsilon_{1}\right)I_{h}$$ (3)

$$I_{hm}^{'}=\beta_{u}\left(T\right)I_{1}I_{m}+\beta_{i}\left(T\right)I_{2}I_{h}-\left(\delta +\delta_{i}+\delta_{u}+\upsilon_{1}\right)I_{hm}$$ (4)

$$R_{m}^{'}=\gamma I_{m}+\tau_{1}\left(1-\delta \right)I_{hm}-\left(\alpha +\upsilon_{1}\right)R_{m}$$ (5)

$$R_{h}^{'}=\omega I_{h}+(1-\tau_{1})\left(1-\delta \right)I_{hm}-\left(\varepsilon +\upsilon_{1}\right)R_{h}$$ (6)

$$R_{hm}^{'}=\delta I_{hm}-\left(\theta +\upsilon_{1}\right)R_{hm}$$ (7)

$$S_{1}^{'}=\Lambda_{1}-\beta_{1}\left(T\right)\left(I_{h}+I_{hm}\right)S_{1}-\gamma_{1}\left(T\right)S_{1}$$ (8)

$$I_{1}^{'}=\beta_{1}\left(T\right)\left(I_{h}+I_{hm}\right)S_{1}-\left(\gamma_{1}\left(T\right)+\alpha_{1}\left(T\right)\right)I_{1}$$ (9)

$$S_{2}^{'}=\Lambda_{2}-\beta_{2}\left(T\right)\left(I_{m}+I_{hm}\right)S_{2}-\gamma_{2}\left(T\right)S_{2}$$ (10)

$$I_{2}^{'}=\beta_{2}\left(T\right)\left(I_{m}+I_{hm}\right)S_{2}-\left(\gamma_{2}\left(T\right)+\alpha_{2}\left(T\right)\right)I_{2}$$ (11)

All temperature variant parameters are given as functions of temperature, T. Human population increase exponentially with a recruitment rate given by $\Lambda_{h}=\Lambda_{r}e^{-\upsilon_{1}\tau }$, where $\Lambda_{r}$ is the maximum per capita birth rate/immigration rate of human individuals, $\upsilon_{1}$ is the natural mortality rate of humans and *τ* is the earliest age at which an individual is infected. Reproduction rates for *Bulinus* and *Biomphalara* snails are respectively, $\Lambda_{1}$ and $\Lambda_{2}$, while the corresponding natural death rates are $\gamma_{1}\left(T\right)$ and $\gamma_{2}\left(T\right)$. The transmissibility of *S. haematobium* and *S. mansoni* is, respectively, $\beta_{u}\left(T\right)$ and $\beta_{i}\left(T\right)$ to humans, $\beta_{1}\left(T\right)$and $\beta_{2}\left(T\right)$ to snails. In humans, *S. mansoni* and *S. haematobium-*related death rates are $\delta_{i}$ and $\delta_{u}$, whereas in *Bulinus* snails and *Biomphalaria*, they are $\alpha_{1}\left(T\right)$and $\alpha_{2}\left(T\right)$. The recovery rates from *S. mansoni*, *S. haematobium* and co-infection are denoted as *γ*, *ω*, and *δ* respectively, while the immunity waning rates are represented by *α*, *ε*, and *θ*. The portion of co-infected individuals who recover from *S. mansoni* is given by the term $\tau_{1}\left(1-\delta \right)$, and the co-infected individuals who recover from *S. haematobium* only are described by $(1-\tau_{1})\left(1-\delta \right)$*.* Hence, the model investigates the comprehensive dynamic effects of a temperature-driven system on schistosomiasis. The model was parameterized using both real experimental temperature variants and non-temperature variants from the literature.

### 2.2. Temperature variant parameters

We collected temperature-dependent data from real experimental and laboratory studies conducted by Mangal et al. [17] for the *Biomphalaria-S. mansoni* system and Kalinda et al. [18] for the *Bulinus-S. haematobium* system. Data from Mangal et al. [17] included temperature-dependent parameters $\beta_{i}\left(T\right)$, $\beta_{2}\left(T\right)$, $\alpha_{2}\left(T\right)$, $\gamma_{2}\left(T\right)$ each with distinct values at 20, 25, 30, and 35 °C (see Table A in S1 text). Similarly, data from Kalinda et al. [18] covered temperature-dependent parameters $\beta_{u}\left(T\right)$, $\beta_{1}\left(T\right)$, $\alpha_{1}\left(T\right)$, $\gamma_{1}\left(T\right)$ at 15, 22, 25.8, 31, and 36 °C (see Table B in S1 text). The data underwent analysis to formulate equations capturing the impact of temperature control on the two systems in actual static environmental conditions. For data analysis, we selected a common temperature range of 20 to 35 °C for both systems. We computed R-squared values and determined the regression equations that produced the highest adjusted value for each temperature-dependent parameter. The regression equations, representing the best fit for the data (curve fitting), are provided in Table 1. Note that Kalinda et al. [18] excluded Schistosomiasis transmission to snails. Yet, statistical comparisons [28–30] suggest higher *S. haematobium* prevalence where both species coexist, therefore, we assumed $\beta_{1}\left(T\right)>\beta_{2}\left(T\right)$.

**Table 1:** Temperature-dependent parameters, symbols, derived curves, ranges, and their sources.

Parameter	*Biomphalaria-S. mansoni* system		Values/day	References
Definition	Symbol	Derived regression equations	range of values	
Transmissibility of schistosomiasis to humans	$\beta_{i}\left(T\right)$	$0.000066T^{2}+0.00259T-0.0488$	0.03469−0.12270	[17]
Transmissibility of schistosomiasis to snails	$\beta_{2}\left(T\right)$	$-0.000009830T^{2}+0.0006148T-0.00826$	0.00032−0.00135	[17]
Schistosomiasis-induced death in snails	$\alpha_{2}\left(T\right)$	$0.00008T^{2}-0.00122T-0.00545$	0.04985−0.07744	[17]
Natural death rate of snails	$\gamma_{2}\left(T\right)$	$0.000112T^{2}-0.00521T+0.0633$	0.00271−0.01815	[17]
	***Bulinus-S. haematobium* system**			
Transmissibility of schistosomiasis to humans	$\beta_{u}\left(T\right)$	0.0063T−0.098	0.0280−0.12250	[18]
Transmissibility of schistosomiasis to snails	$\beta_{1}\left(T\right)$	$1.2\beta_{2}(\text{T)}$	0.00037−0.00162	[18]
Schistosomiasis-induced death in snails	$\alpha_{1}\left(T\right)$	$0.0000732T^{2}-0.00203T+0.01484$	0.00449−0.03346	[18]
Natural death rate of snails	$\gamma_{1}\left(T\right)$	$0.0000794T^{2}-0.002608T+0.02215$	0.00239−0.02814	[18]

The derived equations serve as valuable tools for assessing, comparing, and differentiating between the two genera (*Biomphalaria* and *Bulinus*) under prevailing or anticipated climatic conditions. They provide a means to evaluate transmissibility, survival rates, and mortality rates for both genera within areas characterized by temperatures ranging from 20, 35°C. The applicability of these equations is particularly relevant in Sub-Saharan Africa, specifically East Africa, where *Biomphalaria* and *Bulinus* species are common, and both *S. mansoni* and *S. haematobium* infections are prevalent. The specified temperature ranges correspond to the local climate in the region, making the equations pertinent for studies and assessments in such settings at local or global geographical context.

### 2.3. Temperature invariant parameters

All non-temperature-dependent parameters used in the model were either derived from existing literature or estimated based on expert knowledge (Table 2). For instance, previous observations by Gryseels et al. [4] indicated that schistosomiasis commonly initiates infection in a child at the age of two years. In our model, we represented this age of infection using the parameter τ, set to correspond to 730 days, the two years. Nevertheless, it is important to acknowledge that infants younger than two years can also contract the disease if they come into contact with infected freshwater during activities such as bathing babies. Additionally, the research by Cunin et al. [28], Garba et al. [29], and Nassar et al. [30] reveal a higher occurrence of *S. haematobium* than *S. mansoni* in areas of their coexistence. Therefore, based on this knowledge, we assume that $\delta_{u}>\delta_{i}$ γ>ω>δ, and α>ε>θ. Furthermore, the values of waning immunity *α*, *ε*, and *θ* lie between 0 and 1 because they reflect a proportion or fraction of the original immunity that remains effective at a given point in time. As ε approaches 1, immunity remains strong, while as ε approaches 0, immunity weakens or fades away. Similarly, the values of the recovery rates after treatment (γ,ω) lie between 0 and 1 because they represent a proportion or fraction of the original individuals that remain inffectious at a given point in time. As γ,ω approaches 1, treatment becomes 100% effective, while as γ,ω approaches 0, no individuals recover, and the treatment is not effective. It is important to note that there is no existing epidemic data for either genus to cross-verify aspects such as waning immunity, treatment effectiveness and recovery rates. We have taken values from the literature where available and estimated others within the range of [0, 1].

**Table 2:** Temperature-invariant parameters, symbols, ranges and baseline values, and sources.

Symbol	Definition	Range of values/day	Baseline values	References
$\Lambda_{h}$	Human reproduction rate	100–8,000	100	[31,32]
$\Lambda_{1}$	*Bulinus* snail reproduction rates	100	100	[33]
$\Lambda_{2}$	*Biomphalaria* snail reproduction rates	100	100	[33]
*τ*	Age at first infection in a child	730	730	[4]
${\text{υ}}_{1}$	Human mortality rate	0.0000428–0.0000468	0.0000448	[34]
$\delta_{i}$	S. mansion-human-related death rate	0.000591–0.0039	0.000591	[32,34]
$\delta_{u}$	*S. haematobium*-human-related death rate	0.000591–0.0039	0.0039	[32,34]
*γ*	Recovery rates of *S. mansoni*-infected individuals	0<γ<1	0.050	Estimated
*ω*	Recovery rates of *S. haematobium*-infected individuals	0<ω<1	0.0181	[26]
*δ*	Recovery rates of co-infected individuals from both infections	0<δ<1	0.012	Estimated
$\tau_{1}$	Recovery rates of co-infected individuals from *S. mansoni* infection only	$0<\tau_{1}<1$	0.4	Estimated
*ε*	*Schistosoma haematobium* waning immunity	0<ε<1	0.013	[26]
*α*	*Schistosoma mansoni* waning immunity	0<α<1	0.04	Estimated
*θ*	Co-infection waning rate	0<θ<1	0.009	Estimated

### 2.4. Steady states and the transmissibility of infections

In this study, we examined the stability of infections using both the disease-free equilibrium ($E_{0}$) and the endemic equilibrium ($E_{1}$) and assessed the transmissibility of the infections using the basic reproduction number ($R_{0}$). The disease-free equilibrium represents a state with no active transmission, while the endemic equilibrium signifies ongoing and stable disease transmission within the population. The disease-free equilibrium $E_{0}$ provides a basis for evaluating the effectiveness of control measures, whereas $E_{1}$ offers insights into the persistence and stability of schistosomiasis, while $R_{0}$ quantifies the average number of new infections caused by a single infectious individual in a susceptible population [35]. If $R_{0}>1$, schistosomiasis can emerge, spread, and persist. Conversely, if $R_{0}<1$, the disease-free equilibrium is more likely, as on average, less than one new case is generated during the infectious period. The interplay of the equilibria and $R_{0}$ conditions under climate factors like temperature is vital for shaping public health strategies, providing insight into disease potential, control measure effectiveness, and disease elimination likelihood.

### 2.5. Sensitivity of transmissibility to model parameters

We performed a sensitivity analysis using the partial rank correlation coefficient (PRCC) to assess the impact of individual input parameters on the output variable $R_{0}$. In this analysis, the data is reorganized in ascending order, and the ranks of the variables are substituted. The parameters exhibiting a positive (negative) sign result in an increase (decrease) in the output when they are increased (decreased), and vice versa. The PRCC provides a measure of the monotonic relationship after removing the linear effects of each model parameter while holding all other parameters constant [36]. By employing this approach, one can identify parameters that have the greatest impact and should be the target of interventions. Note that all the simulations methods and the statistical analysis were conducted using the R statistical environment v. 4.0.3 [37].

## 3. Qualitative results

In this study, we formulated a co-dynamic model Eqs. (1)–(11) which can be subdivided into variables *H*, $I_{h}$, $R_{h}$, $S_{1}$, and $I_{1}$ to create a specific sub-model for *S. haematobium* (SH) infection dynamics and variables *H*, $I_{m}$, $R_{m}$, $S_{2}$, and $I_{2}$ to create a specific sub-model for *S. mansoni* (SM) infection dynamics (see the separate sub-model equations in the S1 text). The sub-models facilitated independent analyses of the dynamics of single infections by each *Schistosoma* species. Sections 3.1–3.5 present analyses of the disease-free equilibrium, reproduction number, establishment of endemic equilibria, mutual interaction, and treatment impact for both sub-models and the co-dynamic model. The numerical stability analysis for the equilibrium points in the sub-models and co-dynamics is shown in S1 text. Note that for simplicity, we use the notations; $\beta_{1}\left(T\right)=\beta_{1},\beta_{2}\left(T\right)=\beta_{2}$, $\gamma_{2}\left(T\right)=\gamma_{2}$, $\beta_{u}\left(T\right)=\beta_{u},\beta_{i}\left(T\right)=\beta_{i}$, $\alpha_{1}\left(T\right)=\alpha_{1}$, $\alpha_{2}\left(T\right)=\alpha_{2}$, $\gamma_{1}\left(T\right)=\gamma_{1}$ and $\gamma_{2}\left(T\right)=\gamma_{2}$ in all the sections that follow

### 3.1. *Schistosoma haematobium* (SH) sub-model

To analyze the stability of the SH sub-model, we first established the disease-free equilibrium ($E_{0h}$) and reproduction number ($R_{0h}$) of the *S.haematobium* infection. The SH sub-model has a disease-free equilibrium point given as


$$E_{0h}=(H^{*},I_{h}^{*},\;R_{h}^{*}\;S_{1}^{*};I_{1}^{*})=\left(\frac{\Lambda_{h}}{\upsilon_{1}},0,0,\frac{S_{1}}{\gamma_{1}},0\right)$$

Using the next-generation matrix approach [35,38], we show that


$$F=\left(\begin{array}{cc} 0 & \frac{\beta_{u}\Lambda_{h}}{\upsilon_{1}}\\ \frac{\beta_{1}\Lambda_{1}}{\gamma_{1}} & 0 \end{array}\right),\;V=\left(\begin{array}{cc} \left(\omega +\delta_{u}+\upsilon_{1}\right) & 0\\ 0 & \left(\gamma_{1}+\alpha_{1}\right) \end{array}\right)$$

where *F* is the rate at which new infections arise in one compartment, and *V* is the rate at which people and *Biomphalaria* snails are transferred into that compartment. According to a Jacobian matrix evaluated at $E_{0h}$, $R_{0h}$ is the dominant eigenvalue of $FV^{-1}$ given as


$$R_{0h}=\sqrt{\frac{\beta_{1}\beta_{u}\Lambda_{1}\Lambda_{h}}{\upsilon_{1}\gamma_{1}\left(\gamma_{1}+\alpha_{1}\right)\left(\omega +\delta_{u}+\upsilon_{1}\right)}}$$ (12)

Thus, $R_{0h}$ in equation Eq. (12) depends on temperature *T*, portraying the standard expression of $R_{0h}\left(T\right)$ for new *Schistosoma haematobium* cases, which is influence by the temperature-sensitive parameters $\beta_{1}$, $\beta_{u}$, $\gamma_{1}$, and $\alpha_{1}$. When $R_{0h}<1$ for specific temperature values, the SH sub-model exhibits a disease-free equilibrium, countering infection. Conversely, when $R_{0h}>1$, an endemic equilibrium point $E_{h}$ emerges in the SH sub-model, facilitating infection persistence and establishment. By setting the system of differential equations in the SH sub-model to zero allows for the computation of the endemic equilibrium point $E_{h}=(H^{\prime \prime }$,$I_{h}^{"}$, $R_{h}^{"}$, $S_{1}^{"};I_{1}^{"})$_,_ expressed in terms of $I_{h}$, where


$$H^{\prime \prime }=\frac{\left(\gamma_{1}+\alpha_{1}\right)\left(\left(\varepsilon +\upsilon_{1}\right)\Lambda_{1}+\varepsilon \omega I_{h}^{"}\right)\left(\beta_{1}I_{h}^{"}+\gamma_{1}\right)}{\left(\varepsilon +\upsilon_{1}\right)\left(\gamma_{1}+\alpha_{1}\right)\left(\beta_{1}I_{h}^{"}+\gamma_{1}\right)}\;,\;R_{h}^{"}=\frac{\omega }{\left(\varepsilon +\upsilon_{1}\right)}I_{h}^{"},\;S_{1}^{"}=\frac{\Lambda_{1}}{\left(\beta_{1}I_{h}^{"}+\gamma_{1}\right)}I_{h}^{"},\;I_{1}^{"}=\frac{\beta_{1}\Lambda_{1}I_{h}^{"}}{\left(\gamma_{1}+\alpha_{1}\right)\left(\beta_{1}I_{h}^{"}+\gamma_{1}\right)}I_{h}^{"},$$

By substituting the values of *H*^″^ and $I_{1}^{"}$ into the equation representing *S. haematobium* infected humans ($I_{h}$) from the SH sub-model, we can obtain the solution for $I_{h}^{"}$. The resulting polynomial, given by equation Eq. (13), satisfies the endemic equilibrium of the SH sub-model.


$$\lambda^{3}+a_{2}\lambda^{2}+a_{1}\lambda +a_{0}=0$$ (13)

$$\text{where}a_{0}=-\frac{\gamma_{1}\beta_{u}\beta_{1}\Lambda_{1}\Lambda_{h}}{\beta_{1}\left(\gamma_{1}+\alpha_{1}\right)\left(\omega +\delta_{u}+\upsilon_{1}\right)}\;,\;a_{1}=\frac{\left(\gamma_{1}+\alpha_{1}\right)\left(\omega +\delta_{u}+\upsilon_{1}\right)\left(\gamma_{1}-\beta_{1}\Lambda_{h}\right)-\gamma_{1}\beta_{u}\beta_{1}\Lambda_{1}}{\beta_{1}\left(\gamma_{1}+\alpha_{1}\right)\left(\omega +\delta_{u}+\upsilon_{1}\right)},\;$$

$$a_{2}=\frac{\left(\gamma_{1}+\alpha_{1}\right)\left(\omega +\delta_{u}+\upsilon_{1}\right)\left(\gamma_{1}-\beta_{1}\Lambda_{h}\right)-\left(\gamma_{1}+\alpha_{1}\right)\varepsilon \omega \gamma_{1}\beta_{u}\beta_{1}\Lambda_{1}}{\beta_{1}\gamma_{1}\left(\gamma_{1}+\alpha_{1}\right)\left(\omega +\delta_{u}+\upsilon_{1}\right)}.$$

There is no doubt that $a_{0}<0$, and according to Descartes’ rule of signs [39], if any or both of $a_{1}$ and/or $a_{2},$ at least one positive root results, and therefore an endemic equilibrium exists. Note that the prevalence of endemicity and infection levels across subpopulations fluctuate in response to environmental changes driven by temperature variations.

### 3.2. *Schistosoma mansoni* (SM) sub-model

A disease-free equilibrium point for the SM sub-model is given as


$$E_{0m}=(H^{*},\;I_{m}^{*},\;R_{m}^{*},S_{2}^{*},I_{2}^{*})=\left(\frac{\Lambda_{h}}{\upsilon_{1}},0,0,\frac{S_{2}}{\gamma_{2}},0\right)$$

Similarly, we show that the SM sub-model has a reproduction number $R_{0m}$ given as


$$R_{0m}=\sqrt{\frac{\beta_{2}\beta_{i}\Lambda_{2}\Lambda_{h}}{\upsilon_{1}\gamma_{2}\left(\gamma_{2}+\alpha_{2}\right)\left(\gamma +\delta_{i}+\upsilon_{1}\right)}}$$ (14)

Similarly, as described in Section 3.1, $R_{0m}$ in Eq. (14) reflects temperature-dependent *Schistosoma mansoni* new case influenced by temperature dependent parameters $\beta_{2}$, $\beta_{i}$, $\gamma_{2}$, and $\alpha_{2}$. When $R_{0m}<1$, the SM sub-model reaches a disease-free equilibrium, and such temperature conditions hinders infection. Under favorable temperature conditions, $R_{0m}>1$, indicating the presence of an endemic equilibrium point $E_{h}=(H^{*}$,$I_{m}^{*}$, $R_{m}^{*}$, $S_{2}^{*};I_{2}^{*})$ in the SM sub-model, expressed as follows:


$$H^{*}=\frac{\left(\gamma_{2}+\alpha_{2}\right)\left(\left(\alpha +\upsilon_{1}\right)\Lambda_{2}+\gamma \omega I_{m}^{*}\right)\left(\beta_{2}I_{m}^{*}+\gamma_{2}\right)}{\left(\alpha +\upsilon_{1}\right)\left(\gamma_{2}+\alpha_{2}\right)\left(\beta_{2}I_{m}^{*}+\gamma_{2}\right)},\;R_{m}^{*}=\frac{\gamma }{\left(\alpha +\upsilon_{1}\right)}I_{m}^{*},\;S_{2}^{*}=\frac{\Lambda_{2}}{\left(\beta_{2}I_{m}+\gamma_{2}\right)}I_{m}^{*},\;I_{2}^{*}=\frac{\beta_{2}\Lambda_{2}I_{m}^{*}}{\left(\gamma_{2}+\alpha_{2}\right)\left(\beta_{2}I_{m}^{*}+\gamma_{2}\right)}I_{m}^{*},$$

where the solution for $I_{m}^{*}$, representing *S. mansoni* infected humans in the SM sub-model, is derived by substituting the values of $H^{*}$ and $I_{2}^{"}$ into the equation. The resulting polynomial, described by equation Eq. (15), establishes the endemic equilibrium of the SM sub-model.


$$\lambda^{3}+b_{2}\lambda^{2}+b_{1}\lambda +b_{0}=0$$ (15)

$$\text{where}b_{0}=-\frac{\gamma_{2}\beta_{i}\beta_{2}\Lambda_{2}\Lambda_{h}}{\beta_{2}\left(\gamma_{2}+\alpha_{2}\right)\left(\gamma +\delta_{i}+\upsilon_{1}\right)},\;b_{1}=\frac{\left(\gamma_{2}+\alpha_{2}\right)\left(\omega \gamma +\delta_{i}+\upsilon_{1}\right)\left(\gamma_{2}-\beta_{2}\Lambda_{h}\right)-\gamma_{2}\beta_{i}\beta_{2}\Lambda_{2}}{\beta_{2}\left(\gamma_{2}+\alpha_{2}\right)\left(\gamma +\delta_{i}+\upsilon_{1}\right)},$$

$$b_{2}=\frac{\left(\gamma_{2}+\alpha_{2}\right)\left(\gamma +\delta_{i}+\upsilon_{1}\right)\left(\left(\gamma_{2}-\beta_{2}\Lambda_{h}\right)\right)-\left(\gamma_{2}+\alpha_{2}\right)\gamma \gamma_{2}\beta_{i}\beta_{2}\Lambda_{2}}{\beta_{2}\gamma_{2}\left(\gamma_{2}+\alpha_{2}\right)\left(\gamma +\delta_{i}+\upsilon_{1}\right)}.$$

Thus, it is clear that $b_{0}<0$. Following Descartes’ rule of signs [39], if either or both of $b_{i}>0,i=1,2$, there will be at least one positive root, leading to the existence of an endemic equilibrium. Whereby, changes in temperatures can alter infection prevalence triggered by changes in temperature dependent parameters $\beta_{2}$, $\beta_{i}$, $\gamma_{2}$, and $\alpha_{2}$.

### 3.3. Co-dynamics model

The co-dynamics model in equations Eqs. (1)–(11) has a disease-free equilibrium point given by


$$\begin{array}{c} E_{0}hm=(H^{**},I_{m}^{**},I_{h}^{**},I_{hm}^{**},R_{m}^{**},R_{h}^{**},R_{hm}^{**},S_{1}^{**},I_{1}^{**},S_{2}^{**},I_{2}^{**})\\ =\left(\frac{\Lambda_{h}}{\upsilon_{1}},0,0,0,0,0,0,\frac{\Lambda_{1}}{\gamma_{1}},0,\frac{\Lambda_{2}}{\gamma_{2}},0\right) \end{array}$$

Similarly, linearization of the co-dynamic model at $E_{0hm}$ can be ascertained where,


$$F=\left(\begin{array}{ccccc} 0 & 0 & 0 & 0 & \frac{\beta_{i}\Lambda_{h}}{\upsilon_{1}}\\ 0 & 0 & 0 & \frac{\beta_{u}\Lambda_{h}}{\upsilon_{1}} & 0\\ 0 & 0 & 0 & 0 & 0\\ 0 & \frac{\beta_{1}\Lambda_{1}}{\gamma_{1}} & \frac{\beta_{1}\Lambda_{1}}{\gamma_{1}} & 0 & 0\\ \frac{\beta_{2}\Lambda_{2}}{\gamma_{2}} & 0 & \frac{\beta_{2}\Lambda_{2}}{\gamma_{2}} & 0 & 0 \end{array}\right)$$

$$V=\left(\begin{array}{ccccc} \left(\gamma +\delta_{i}+\upsilon_{1}\right) & 0 & 0 & 0 & 0\\ 0 & \left(\omega +\delta_{u}+\upsilon_{1}\right) & 0 & 0 & 0\\ 0 & 0 & \left(\delta +\delta_{i}+\delta_{u}+\upsilon_{1}\right) & 0 & 0\\ 0 & 0 & 0 & \left(\gamma_{1}+\alpha_{1}\right) & 0\\ 0 & 0 & 0 & 0 & \left(\gamma_{2}+\alpha_{2}\right) \end{array}\right)$$

Similarly, we can get the next generation matrix and $R_{0hm}$ for the co-dynamics model:


$$FV^{-1}=\left(\begin{array}{ccccc} 0 & 0 & 0 & 0 & \frac{\beta_{i}\Lambda_{h}}{\upsilon_{1}\left(\gamma_{2}+\alpha_{2}\right)}\\ 0 & 0 & 0 & \frac{\beta_{u}\Lambda_{h}}{\upsilon_{1}\left(\gamma_{1}+\alpha_{1}\right)} & 0\\ 0 & 0 & 0 & 0 & 0\\ 0 & \frac{\beta_{1}\Lambda_{1}}{\gamma_{1}\left(\omega +\delta_{u}+\upsilon_{1}\right)} & \frac{\beta_{1}\Lambda_{1}}{\gamma_{1}\left(\delta +\delta_{i}+\delta_{u}+\upsilon_{1}\right)} & 0 & 0\\ \frac{\beta_{2}\Lambda_{2}}{\gamma_{2}\left(\gamma +\delta_{i}+\upsilon_{1}\right)} & 0 & \frac{\beta_{2}\Lambda_{2}}{\gamma_{2}\left(\delta +\delta_{i}+\delta_{u}+\upsilon_{1}\right)} & 0 & 0 \end{array}\right)$$

There are two eigenvalues that could both be the largest/dominant depending on the parameter values [38,40];


$$R_{0h}^{2}=\frac{\beta_{1}\beta_{u}\Lambda_{1}\Lambda_{h}}{\upsilon_{1}\gamma_{1}\left(\gamma_{1}+\alpha_{1}\right)\left(\omega +\delta_{u}+\upsilon_{1}\right)},\text{and}R_{0m}^{2}=\frac{\beta_{2}\beta_{i}\Lambda_{2}\Lambda_{h}}{\upsilon_{1}\gamma_{2}\left(\gamma_{2}+\alpha_{2}\right)\left(\gamma +\delta_{i}+\upsilon_{1}\right)}$$

Consequently, the basic reproductive number is the square root of the largest of these two eigenvalues.


$$R_{0hm}=max\left\{R_{0h},R_{0m}\right\}$$

Thus, the emergence of *Schistosoma* co-infection cases hinges on the influence of temperature on either $R_{0h}$ or $R_{0m}$. The subsequent Theorem 1 establishes this dependency.

Theorem 1: The disease free equilibrium $E_{0hm}$ in co-dynamic model is locally asymptotically stable whenever $R_{0hm}<1$ and unstable otherwise (see the proof in S1 text)

### 3.4. Mutual interactions: Impact of *S. haematobium* on *S. mansoni* and vice versa

This section explores mutual effects of *S. haematobium* and *S. mansoni* by expressing their reproduction numbers bidirectionally. This approach enables us to explore the relationship between the reproduction numbers of the two infections and gain insights into their mutual interactions. We begin by expressing $R_{0h}$ in equation Eq. (12) in terms of $R_{0m}$ given in equation Eq. (14), where we solve for $\upsilon_{1}$in $R_{0m}$ and substitute in $R_{0h}$, to get


$$\upsilon_{1}=\frac{-q_{1}R_{0m}+\sqrt{{\left(q_{1}R_{0m}\right)}^{2}+4p_{1}r_{1}}}{2p_{1}R_{0m}}$$

where $p_{1}=\gamma_{2}\left(\gamma_{2}+\alpha_{2}\right)$, $q_{1}=\gamma_{2}\left(\gamma_{2}+\alpha_{2}\right)\left(\gamma +\delta_{i}\right)$, and $r_{1}=\beta_{i}\beta_{2}\Lambda_{2}\Lambda_{h}$. Substituting $\upsilon_{1}$ into expression for $R_{0h}$, we obtain


$$R_{0h}=\sqrt{\left(\frac{2r_{2}p_{1}^{2}}{q_{1}\left(q_{1}p_{2}-p_{1}q_{2}\right)R_{0m}+\left(p_{1}q_{2}-q_{1}p_{2}\right)\sqrt{{\left(q_{1}R_{0m}\right)}^{2}+4p_{1}r_{1}}+2p_{1}p_{2}r_{1}R_{0m}^{-1}}\right)}$$ (16)

where $p_{2}=\gamma_{1}\left(\gamma_{1}+\alpha_{1}\right)$, $q_{2}=\gamma_{1}\left(\gamma_{1}+\alpha_{1}\right)\left(\omega +\delta_{u}\right)$, and $r_{2}=\beta_{u}\beta_{1}\Lambda_{1}\Lambda_{h}$.

Similarly, expressing $\upsilon_{1}$in terms of $R_{0h}$ leads to


$$\upsilon_{1}=\frac{-q_{2}R_{0h}+\sqrt{{\left(q_{2}R_{0h}\right)}^{2}+4p_{2}r_{2}}}{2p_{2}R_{0h}}$$

Substituting $\upsilon_{1}$ into expression for $R_{0m}$, we get


$$R_{0m}=\sqrt{\left(\frac{2r_{1}p_{2}^{2}}{q_{2}\left(q_{2}p_{1}-p_{2}q_{1}\right)R_{0h}+\left(p_{2}q_{1}-q_{2}p_{1}\right)\sqrt{{\left(q_{2}R_{0h}\right)}^{2}+4p_{2}r_{2}}+2p_{1}p_{2}r_{2}R_{0h}^{-1}}\right)}$$ (17)

Partial derivatives of $R_{0h}$ in equation Eq. (16) and $R_{0m}$ in equation Eq. (17), determine the co-infection impact of *S. mansoni* on *S. haematobium* and *haematobium* on *S. mansoni*, respectively, in a population. By partially differentiating $R_{0h}$ in Eq. (16) with respect to $R_{0h}$, we obtain


$$\frac{\partial R_{oh}}{\partial R_{om}}=\frac{\sqrt{2r_{2}p_{1}^{2}}\left\{q_{1}(q_{2}p_{1}-p_{2}q_{1})+2q_{1}(q_{2}p_{1}-p_{2}q_{1})R_{om}{\left(\sqrt{{\left(q_{1}R_{om}\right)}^{2}+4p_{1}r_{1}}\right)}^{-1}-2p_{1}p_{2}r_{1}R_{om}^{-2}\right\}}{{\left\{q_{1}(q_{2}p_{1}-p_{2}q_{1})R_{om}+(p_{2}q_{1}-q_{2}p_{1})\sqrt{{\left(q_{1}R_{om}\right)}^{2}+4p_{1}r_{1}+4p_{1}r_{1}}+2p_{1}r_{1}R_{om}^{-1}+2p_{1}p_{2}r_{1}R_{om}^{-1}\right\}}^{3/2}}$$ (18)

Similarly, by partially differentiating $R_{0m}$ in equation Eq. (17) with respect to $R_{0h}$, we are able to derive


$$\frac{\partial R_{oh}}{\partial R_{om}}=\frac{\sqrt{2r_{2}p_{1}^{2}}\left\{q_{2}(q_{1}p_{2}-p_{1}q_{2})+2q_{2}(q_{1}p_{2}-p_{1}q_{2})R_{oh}{\left(\sqrt{{\left(q_{2}R_{oh}\right)}^{2}+4p_{2}r_{2}}\right)}^{-1}-2p_{1}p_{2}r_{2}R_{om}^{-2}\right\}}{{\left\{q_{2}(q_{2}p_{1}-p_{2}q_{1})R_{oh}+(p_{1}q_{2}-q_{1}p_{2})\sqrt{{\left(q_{2}R_{oh}\right)}^{2}+4p_{2}r_{2}}+2p_{2}r_{2}R_{oh}^{-1}+2p_{1}p_{2}r_{2}R_{oh}^{-1}\right\}}^{3/2}}$$ (19)

The partial derivatives in equations Eq. (18) and Eq. (19) reveal distinct scenarios. Equation Eq. (18) determines the temperature-driven influence of *S. haematobium* on *S. mansoni*. If $\left(\partial R_{0h}/\partial R_{0m}\right)>0$ under specific environmental conditions, an increase in *S. mansoni* cases boosts *S. haematobium* infection, favoring both infections. Conversely, $\left(\partial R_{0h}/\partial R_{0m}\right)=0$ signifies no significant impact of *S. mansoni* changes on *S. haematobium* transmission. If $\left(\partial R_{0h}/\partial R_{0m}\right)<0$, an increase in *S. mansoni* cases reduces *S. haematobium* cases, negatively affecting *S. haematobium* but favoring emerging *S. mansoni* cases. Equation Eq. (19) similarly assesses the temperature-dependent impact of *S. mansoni* on *S. haematobium*.

### 3.5. Impacts on treatment inferred from the recovery rate

Furthermore, our model assumes that infected individuals recover due to treatment. Consequently, in individuals co-infected with both *S. mansoni* and *S. haematobium*, the effect of treatment with praziquantel is likely to be a reduction of worm burdens for both forms of schistosomes in the human body. The effect can be determined by evaluating the partial derivatives of $R_{0hm}=max\left\{R_{0h},R_{0m}\right\}$with respect to the recovery rate (*γ*) of individuals from *S. mansoni* and (*ω*) of individuals from *S. haematobium*.. For example, if $R_{0h}>R_{0m}$ then $R_{0hm}=R_{0h}$, the derivation yields insights into.


$$\frac{\partial R_{0hm}}{\partial \gamma \partial \omega }=\sqrt{2r_{2}p_{1}^{2}}\left(\frac{D_{2}q_{1}p_{1}\left\{R_{0m}-{\left[{\left(q_{1}R_{0m}\right)}^{2}+4p_{1}r_{1}\right]}^{-1/2}\right\}+3D_{1}p_{1}\left\{q_{1}R_{0m}-{\left[{\left(q_{1}R_{0m}\right)}^{2}+4p_{1}r_{1}\right]}^{-1/2}\right\}}{4{\left(D_{2}\right)}^{2}}\right)$$ (20)

$$D_{1}=q_{1}\left(p_{1}q_{2}-2q_{1}p_{2}\right)R_{0m}+p_{2}\sqrt{{\left(q_{1}R_{0m}\right)}^{2}+4p_{1}r_{1}}+q_{1}\left(q_{1}p_{2}-p_{1}q_{2}\right){\left[{\left(q_{1}R_{0m}\right)}^{2}+4p_{1}r_{1}\right]}^{-1/2}$$

$$D_{2}=2{\left(q_{1}\left(q_{1}p_{2}-p_{1}q_{2}\right)R_{0m}+\left(p_{1}q_{2}-q_{1}p_{2}\right)\sqrt{{\left(q_{1}R_{0m}\right)}^{2}+4p_{1}r_{1}}+2p_{1}p_{2}r_{1}R_{0m}^{-1}\right)}^{3}$$

Thus depending on environmental conditions, the cost-effectiveness of treating both *S. mansoni* and *S. haematobium* in a mixed infection model holds different implications depending on the sign of equation Eq. (20). A negative value, $\text{Eq}.\left(20\right)<0$ indicates a potential synergy, reducing the transmission potential against a mixed infection. A value of zero, $\text{Eq}.\left(20\right)=0$ implies no substantial impact of treatment on schistosomiasis co-infection dynamics. Conversely, a positive value, $\text{Eq}.\left(20\right)>0$ suggests a potential increase in the transmission potential of a mixed infection. This suggests an an antagonistic effect or heightened risk of transmission when *S. mansoni* and *S. haematobium* are treated with a single praziquantel treaetment alone. Similarly, the impact of treatment can be assessed through the recovery rate *γ* for individuals infected with *S. mansoni*, using the partial derivative $\partial R_{0m}/\partial \gamma$. Likewise, the impact of treatment on individuals infected with *S. haematobium*   can be analyzed through the recovery rate *ω* using the partial derivative $\partial R_{0h}/\partial \omega$. These analytical insights help in formulating an optimal treatment strategy based on specific seasonal or monthly temperature variations. However, it is important to note that the effectiveness of treatment may be influenced by other factors such as the stage of each infection, the severity of the disease, and individual variations in response to the drug.

## 4. Numerical simulations

**Fig 1:**
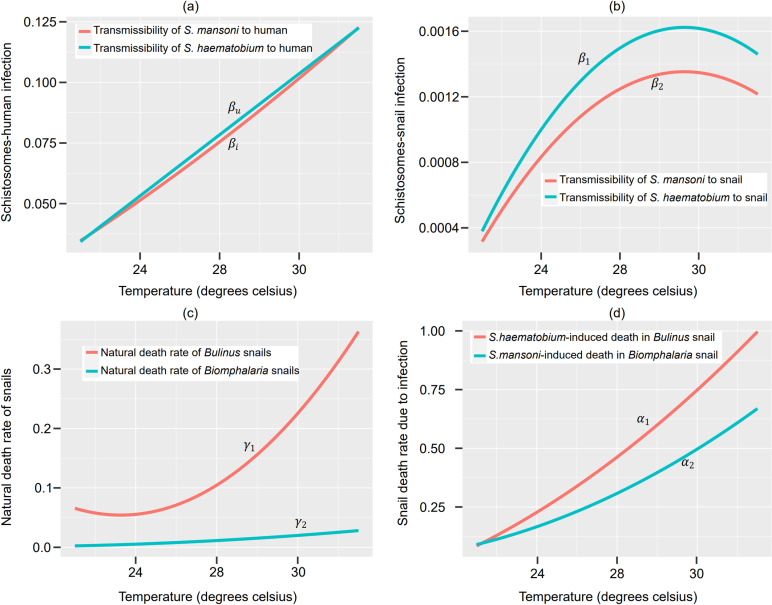
Temperature-dependent model parameters in the life history of *Schistosoma* species between human and intermediate host genera.

Based on the parameter values in Table 1 and Table 2, we simulate the dynamics of co-infection between *S. haematobium* and *S. mansoni*. The simulation outcomes demonstrate a linear relationship between the transmissibility rate of *S. haematobium* ($\beta_{u}$) and *S. mansoni* ($\beta_{i}$) to humans (Fig. 1a). These results also reveal a similar pattern of changes in temperature for both infections. However, the transmissibility of *S. mansoni* to *Biomphalaria* snails ($\beta_{2}$) displays greater sensitivity to temperature variations compared to the transmissibility of *S. haematobium* to *Bulinus* snails ($\beta_{1}$), as shown in Fig. 1b. Furthermore, it is observed that the natural death rate ($\gamma_{1}$) and *S. haematobium*-induced death ($\alpha_{1}$) in *Bulinus* snails exhibit higher sensitivity to temperature changes than the natural death rate ($\gamma_{2}$) and *S. mansoni*-induced death rate ($\alpha_{2}$) in *Biomphalaria* snails as depicted in Fig. 1c and Fig. 1d. This indicates that the two species respond differently to environmental temperature variations, leading to distinct impacts on the dissemination of the single and mixed infections within the populations.

**Fig 2:**
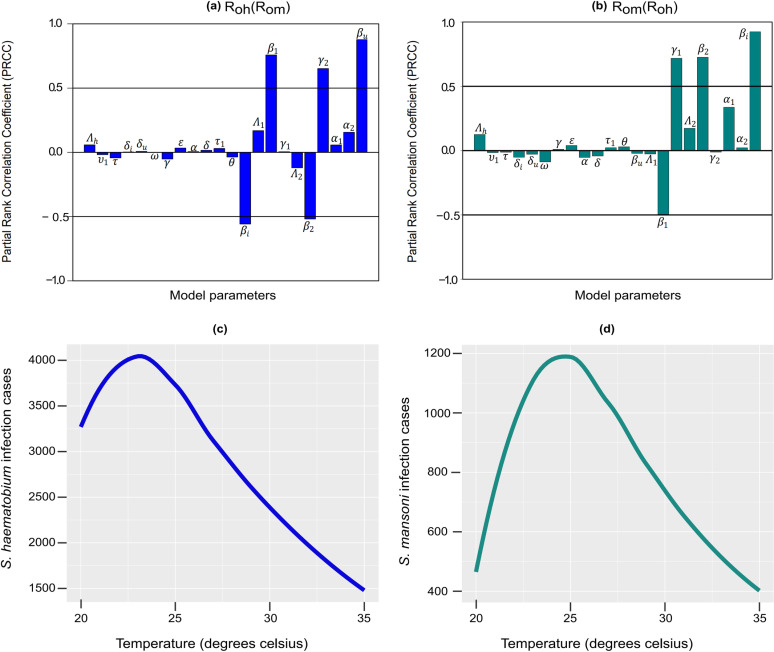
Impacts of individual input parameters on the output variables (a) $R_{0h}$ as a function of $R_{0m}$ and (b) $R_{0m}$ as a function of $R_{0h}$. (c) Variations in *S. haematobium* infection cases, and (d) *S. mansoni* infection cases due to changes in temperature-dependent model parameters (note different scales for infection cases for *S. haematobium and S. mansoni* in (c) and (d).

In the sensitivity analysis, we further identify the most sensitive and significant parameters in $R_{0h}$ and examine their mutual interactions, which are influenced by the interplay of other parameters in the co-dynamics model. Specifically, we use $\left|\text{PRCC}\right|\geq 0.5$ as the threshold indicated by the horizontal lines in Fig. 2a and Fig. 2b. Our findings show that the reproduction number of *S. haematobium* ($R_{0h}$) is highly sensitive to changes in the transmissibility of *S. haematobium* to humans ($\beta_{u}$), the transmissibility of *S. haematobium* to snails ($\beta_{1}$), the natural death rate of *Biomphalaria* snails ($\gamma_{2}$), the transmissibility of *S. mansoni* to humans ($\beta_{i}$), and the transmissibility of *S. mansoni* to snails ($\beta_{2}$), in that order as depicted in Fig. 2a. Similarly, the reproduction number of *S. mansoni* ($R_{0m}$) exhibits sensitivity to changes in the transmissibility of *S. mansoni* to humans ($\beta_{i}$), the natural death rate of *Bulinus* snails ($\gamma_{1}$), the transmissibility of *S. mansoni* to snails ($\beta_{2}$), and the transmissibility of *S. haematobium* to snails ($\beta_{1}$), in that order, as depicted in Fig. 2b. These parameters stand out as crucial and could serve as potential targets for controlling both infections. In general, within an endemically co-infected community, $R_{0h}$ shows greater sensitivity to S*. mansoni* parameters, whereas $R_{0m}$demonstrates lesser sensitivity to *S. haematobium* parameters. This indicates that changes in *S. mansoni* infections, $R_{0m}$ have a lesser impact on transmission of haematobium, compared to the reverse scenario. Thus, it can be inferred that *S. haematobium* has a positive impact on *S. mansoni* dynamics, suggesting that *S. haematobium* may modulate the immune response to increase susceptibility. Consequently, $R_{0h}>R_{0m}$ in endemically co-infected communities, especially when considering how temperature-dependent parameters vary simultaneously due to climate change in such regions; see Fig. 2c and Fig. 2d. Furthermore, regions or seasons characterized by temperature fluctuations of approximately 23–26ºC tend to exhibit the highest number of infection cases from both species (Fig 2c, 2d). However, in the presence of temperature fluctuations above 26ºC degrees, a notable decrease in the number of infections and burden is observed; see Fig. 2c and Fig. 2d. This phenomenon can likely be attributed to a higher mortality rate among the intermediate hosts (Fig. 1c, Fig. 1d) and a concurrent decline in their infection rates (Fig. 1b).

Furthermore, we utilized the temperature-dependent parameter curves (in Table 1) to derive temperature variant parameter values at 20°C, 25°C, and 35°C, representing distinct seasons and geographical regions with potentially diverse climatic conditions, as outlined in Table 3. Subsequently, simulations of the co-dynamic model Eqs. (1)–(11) were conducted to depict the progression of single and mixed infections across a 5-year period.

**Table 3:** Temperature-dependent parameter values under stable temperatures conditions.

	$\beta_{1}$	$\beta_{u}$	$\beta_{1}$	$\beta_{2}$	$\gamma_{1}$	$\gamma_{2}$	$\alpha_{1}$	$\alpha_{2}$
20°C	0.0294	0.0280	$0.000125\beta_{i}$	0.000104	0.00175	0.0039	0.00352	0.00390.00215
25°C	0.0572	0.0595	0.00116	0.00097	0.006575	0.00305	0.00305	0.01405
35°C	0.01227	0.1225	0.0014590.014050.02814	0.001216	0.02814	0.01815	0.03346	0.04985

**Fig 3:**
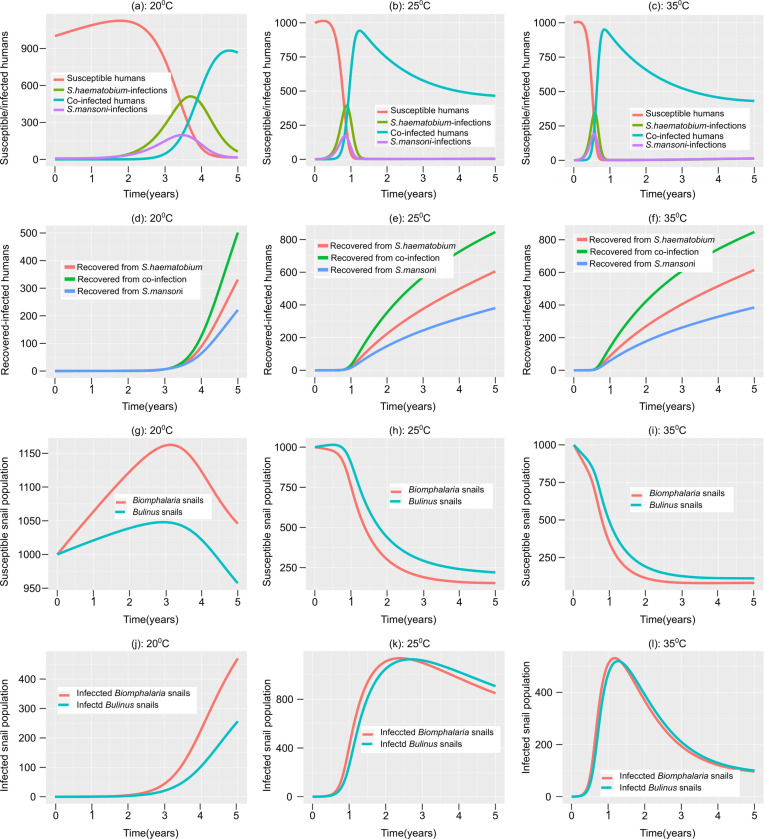
Temperature effects on populations of *S. haematobium* and *S. mansoni* infections. (a-c) Susceptible and infected human populations; (d-f) Recovered human population; (g-i) Susceptible snail population; (j-l) Infected snail population.

Our study underscores temperature-dependent variations in infection levels among hosts and individuals, particularly at moderate temperatures (20°C and 25°C) compared to higher temperatures (35°C), resulting in decreased infections rates (Fig. 3a–c). Notably, we observe different temperature-related impacts on dissemination. *S. haematobium* exhibits higher dissemination rates at 20°C in the short term (1–3 years) but faces a higher co-infection burden in the long term (Fig. 3a). Conversely, rapid dissemination occurs in a shorter time with increased co-infection cases at 25°C and 35°C (Fig. 3c–b). The recovery rates also vary with temperature, with more individuals recovering from *S. haematobium* than *S. mansoni* or mixed infections (Fig. 3d–f). This underscores variations in response to treatment and recovery patterns across different infection types, with recovery being lower at 20°C but higher at 25°C and 35°C (Fig. 3d–f). Moreover, our simulations reveal that despite an increase in the *Biomphalaria* snail population, these snails exhibit higher susceptibility to infection compared to *Bulinus* snails at 25°C, resulting in more *Biomphalaria* infections than *Bulinus* cases (Fig. 3g–j). Additionally, *Biomphalaria* snails show a more pronounced decrease in population at temperatures between 25°C and 35°C compared to *Bulinus* snails (Fig. 3h–l), suggesting potential differences in sensitivity and resistance to temperature changes between these snail species. These variations arise from the non-linear effects of temperature on *Schistosoma* traits within their life history.

## 5. Discussion

Climate-induced challenges like drought, population displacements, poverty, and poor sanitation hinder disease control in endemic regions such as Sub-Saharan Africa, where schistosomiasis is prevalent [1–4]. Integrating climatic variability into complex multi-host disease models, like schistosomiasis, is challenging and debatable. The scarcity of climatic experimental and epidemiological data for model parameterization adds to the complexity. Nevertheless, two existing laboratory studies providing comprehensive temperature-related data in the literature served as inspiration for this study, see [17,18]. This study employs a mathematical model to comprehensively explore the co-dynamics between *S. mansoni* and *S. haematobium*, revealing temperature-related variations in their transmission dynamics, interactions, and implications for both single and mixed schistosomiasis infections. Standard mathematical techniques are applied to calculate and present theoretical properties of the single species sub-models and co-dynamic model, including disease-free and endemic states as functions of temperature. The study derives standard expressions for reproduction numbers ($R_{0}$s) under static environmental conditions with temperature-dependent entities, while assessing the local and global stability of equilibria associated with single species sub-models and co-dynamic model, offering detailed biological interpretations. The reproduction numbers are used to demonstrate mutual interaction effects between both species and evaluate the impact of infection on each other. The study also examines the impact of treatment inferred from the recovery rate, establishing temperature conditions for disease-free and disease prevalence. The theoretical quantitative framework provides analytical insights with user-friendly expressions of $R_{0}$s, aiding in guiding disease control strategies and investigating the contribution of each model parameter to disease spread. For example, it assists in determining an appropriate treatment strategy based on specific seasonal or monthly temperature conditions.

Parameterized with temperature variant and invariant data, our model numerical simulation shows that *S. mansoni* is more sensitive to temperature during transmission to *Biomphalaria* snails than *S. haematobium* to *Bulinus* snails. Contrary, *S. haematobium* exhibits higher sensitivity to temperature in transmission to the human compared to *S. mansoni*. Additionally, Moderate temperatures (20°C and 25°C) increase human infection levels, while higher temperatures (35°C) reduce incidence. Recovery rates of both single and co-infected individuals rise with temperature, favoring *S. haematobium* than *S. mansoni* and mixed infection, suggesting that temperature variations significantly impact the efficacy of schistosomiasis treatment protocols. In addition, co-infections often present overlapping symptoms and complications, complicating the process of accurately distinguishing and effectively treating each infection. The model offers a theoretical framework that simulates these interactions, suggesting that aligning treatment interventions with specific temperature regimes could improve their effectiveness.

Moreover, parameters influencing reproduction numbers underscore a positive influence of *S. haematobium* on *S. mansoni* dynamics. *Biomphalaria* snails are more susceptible than *Bulinus*, with varied temperature impacts on their populations, i.e., temperatures less than 25°C seem favorable while temperatures above 26ºC result in a significant decrease in their population. The variations in schistosomiasis transmissibility between humans and intermediate host snails are primarily associated with temperature-dependent parameters. These findings have significant public health implications, recommending tailored seasonal and timely treatment strategies.

Our findings are consistent with previous studies that demonstrated that transmission traits in the life cycle of the *Schistosoma* species exhibit distinct patterns under different temperature conditions [7,17,18,34,41]. In contrast, the experiment conducted by He and Ramaswamy [42] demonstrated that *S. mansoni* and *S. haematobium* larvae can pass through human skin indistinguishably, resulting in no differences in transmissions. However, this study did not consider temperature fluctuations, thus our results underscores the importance of considering temperature control over the temperature sensitive stages of *Schistosoma* life cycle to unravel the differences, and interactions between closely related single and mixed species infections. For instance, our model revealed the role of *S. haematobium* in mutually increasing susceptibility to *S. mansoni* co-infections. This result is supported by the studies that highlight the impact of *S. haematobium* on the local genital tract and the global immune system [1,12]. Furthermore, earlier studies, including Mbabazi et al. [43], have also established its association with other diseases such as HIV. The cumulative evidence suggests that the effects of *S. haematobium* are not confined to the local site of infection but have systemic consequences. This necessitates a comprehensive approach to the management of *Schistosoma* species, their potential mutual interactions and impacts on co-infections and other diseases such as HIV based on the environmental conditions.

Moreover, the results reveal distinct impacts of temperature on the dynamics of *S. haematobium*-*Bulinus* and *S. mansoni-Biomphalaria* infection. Generally, the latter system exhibits a lower sensitivity to temperature variations, indicating a lower risk of outbreak and fewer infection cases. These findings are consistent with field studies, including Cunin et al. [28], Garba et al. [29], and Nassar et al. [30], which consistently report a higher prevalence of *S. haematobium* compared to *S. mansoni* in areas where both species coexist. Specifically, our study supports prior observations that populations, including both individuals and intermediate hosts, exhibit higher infection levels with *S. haematobium* or *S. mansoni* at moderate temperatures (20°C and 25°C) compared to 35°C, where infections notably decrease [7,17,18,34,41]. The low infection levels indicate restricted schistosomiasis activities, higher mortality rates among intermediate hosts, and notably decreased transmissibility, particularly at 35°C. This consensus underscores the role of temperature in shaping the dynamics of *Schistosoma* species infections in diverse environments and populations. For example, temperature variation in different endemic areas can be a possible explanation for reported cases of low cure rates, and drug resistance, due to persistent transmission patterns and increased re-infection rates [15]. Therefore, the impact of temperature extends beyond schistosomiasis, as it has been shown to shape disease dynamics and stability in various ecological systems [44–47]. Therefore, temperature-dependent models are effective tools for predicting disease patterns in regions where the specified temperature ranges align with the local climate. This applicability is particularly notable in East Africa, where typical temperature ranges, [20,35] °C coincide with the prevalence of *Biomphalaria* and *Bulinus* species, and both *S. mansoni* and *S. haematobium* infections are widespread [4,22,47]. Additionally, the range of demographic parameters $\Lambda_{h}$, $\Lambda_{1}$, *τ*, and ${\text{υ}}_{1}$ align with the rates observed in East Africa, as evident in Tabo et al. [47].

## 6. Limitations and outlook

Although our model remains robust and provides valuable insights, there are some limitations. Firstly, the parameter values employed in our model, reflecting the biological aspects and real-life scenarios of schistosomiasis transmission, were drawn from published literature. This introduces potential inconsistencies and variability within the data collected under diverse conditions, leading to uncertainties in our model results. Secondly, using estimated baseline values introduces potential biases, such as systematic errors and reduced generalizability. Thirdly, it is essential to acknowledge the current absence of real-life epidemiological data for the two infection systems to cross-verify and validate our model. To address this, a crucial step in the future involves applying the model in a schistosomiasis endemic region with established local climates and healthcare or treatment data. This application should encompass a comparison of predicted endemic states with available real-epidemiological data on *S. mansoni* and *S. haematobium* infections. Additionally, future research could further refine our understanding by incorporating human worm burden dynamics and integrating optimal control strategies. These endeavors aim to determine effective means of infection control and represent essential avenues for enhancing our comprehension of schistosomiasis.

## 7. Conclusions

In light of our findings in this study, recognizing the temperature-dependent impact on reproduction numbers underscores the need to integrate temperature into models for predicting and managing schistosomiasis dynamics. Public health and policymakers should implement targeted control strategies, considering seasonal variations in sensitive parameters like snail/human transmissibility and snail natural death rates. Targeting snail control during seasons with temperatures below 25°C to capitalize on increased susceptibility is a strategic approach. It is imperative to monitor and adapt treatment protocols, considering temperature-dependent recovery rates, for enhanced overall treatment effectiveness. For instance, interventions during seasons around 25–35°C, where higher recovery rates are observed, may yield better results. Empowering communities to implement preventive measures during specific temperature conditions can further bolster schistosomiasis control initiatives. Our mathematical model provides a robust framework for understanding the interplay between temperature and various forms of schistosomiasis transmission dynamics. Functioning as a quantitative framework, it offers a reasonable approximation with baseline parameter values, thereby enriching our comprehension of the impact of temperature, and the timing of interventions. Thus, our study strengthens the One Health approach by integrating human and animal (IH snail) health strategies with environmental factors and seasonal variations to optimize schistosomiasis control. The study also shows how the model can be applied in different regions with similar climates.

## Supporting information

S1 text Tables showing the parameters that depend on temperature for the *Biomphalaria*-*S. mansoni* and *Bulinus-S. haematobium* systems. Analysis of the stability of equilibria for each system and their co-infection, both locally and globally.
(DOCX)
