# Supplementary material for: Modelling Temperature-dependent Schistosomiasis Dynamics for Single and Co-infections with S. mansoni and S. haematobium
Source: PLoS One. 2025 Mar 13;20(3):e0318720. doi: 10.1371/journal.pone.0318720 (PMC11906091; doi:10.1371/journal.pone.0318720)
Supplement: S1 text — Analysis of the stability of equilibria for each system and their co-infection, both locally and globally. (DOCX) [file pone.0318720.s001.docx]

**Supplementary information**

**Modelling Temperature-dependent Schistosomiasis Dynamics for Single and Co-infections with *S. mansoni* and *S. haematobium***

**Zadoki Tabo^1,2,3*^, Lutz Breuer^2,3^ & Christian Albrecht^1^**

^1^Department of Animal Ecology and Systematics, Justus Liebig University Giessen, Heinrich-Buff-Ring 26 (iFZ), 35392 Giessen, Germany.

^2^Department of Landscape Ecology and Resource Management, Justus Liebig University Giessen, Heinrich-Buff-Ring 26 (iFZ), 35392 Giessen, Germany.

^3^Centre for International Development and Environmental Research (ZEU), Justus Liebig University Giessen, Senckenbergstrasse 3, 35390 Giessen, Germany.

**^*^**Corresponding Author

E-mail: [Tabo.Zadoki@umwelt.uni-giessen.de](mailto:Tabo.Zadoki@umwelt.uni-giessen.de) (ZT)

**Table A:** Temperature-dependent parameters from Mangal et al. [[1](#Srf1)]

| parameter | Definition | 20ºC | 25ºC | 30ºC | 35ºC |
| --- | --- | --- | --- | --- | --- |
| $\beta_{i}$ | Transmissibility of Schistosomiasis to humans | 0.028 | 0.059 | 0.092 | 0.122 |
| $\beta_{2}$ | Transmissibility of Schistosomiasis to snails | 0.000127 | 0.000091 | 0.0014 | 0.0012 |
| $\gamma_{2}$ | Natural death rate of snails | 0.004 | 0.003 | 0.008 | 0.0182 |
| $\alpha_{2}$ | Schistosomiasis-induced death in snails | 0.002 | 0.0145 | 0.0295 | 0.05 |

**Table B:** Temperature-dependent parameters from Kalinda et al. [[2](#Srf2)]

| parameter | Definition | 15ºC | 22ºC | 25.8ºC | 31ºC | 36ºC |
| --- | --- | --- | --- | --- | --- | --- |
| $\beta_{u}$ | Transmissibility of Schistosomiasis to humans | 0.00638 | 0.0353 | 0.06433 | 0.09753 | 0.12825 |
| $\gamma_{1}$ | Natural death rate of snails | 0.00049 | 0.00434 | 0.00763 | 0.01635 | 0.0318 |
| $\alpha_{1}$ | Schistosomiasis-induced death in snails | 0.00122 | 0.004505 | 0.011989 | 0.022717 | 0.036391 |

**Analysis of the stability of equilibria**

1. **SH sub-model**

The following equations represent the SH sub model

| $H^{'}=\Lambda_{h}+\varepsilon R_{h}-\beta_{u}I_{1}H-\upsilon_{1}H$ |  |
| --- | --- |
| $I_{h}^{'}=\beta_{u}I_{1}H-\left( \omega+\delta_{u}+\upsilon_{1} \right)I_{h}$ |  |
| $R_{h}^{'}=\omega I_{h}-\left( \varepsilon+\upsilon_{1} \right)R_{h}$ | (B.1) |
| $S_{1}^{'}=\Lambda_{1}-\beta_{1}I_{h}S_{1}-\gamma_{1}S_{1}$ |  |
| $I_{1}^{'}=\beta_{1}I_{h}S_{1}-(\gamma_{1}+\alpha_{1})I_{1}$ |  |

**1.1 Local stability of the SH sub-model**

Theorem 2: *The SH sub-model in* [Eq. (B.1)](#eqB1)*,* *at disease free* $E_{0h}$*, is locally asymptotically stable if* $R_{0h}<1$*, otherwise unstable for* $R_{0h}>1$*.*

**Proof**: The SH sub-model [Eq. (B.1)](#eqB1) has the following Jacobian matrix at $E_{0h}$

$J\left( E_{0h} \right)=\left( \begin{matrix} -\upsilon_{1} & 0 & \varepsilon& 0 & \frac{{-\beta_{u}\Lambda}_{h}}{\upsilon_{1}} \\ 0 & -\left( \omega+\delta_{u}+\upsilon_{1} \right) & 0 & 0 & \frac{{\beta_{u}\Lambda}_{h}}{\upsilon_{1}} \\ 0 & \omega& -\left( \varepsilon+\upsilon_{1} \right) & 0 & 0 \\ 0 & \frac{{-\beta_{1}\Lambda}_{1}}{\gamma_{1}} & 0 & -\gamma_{1} & 0 \\ 0 & \frac{{\beta_{1}\Lambda}_{1}}{\gamma_{1}} & 0 & 0 & -(\gamma_{1}+\alpha_{1}) \end{matrix} \right)$ (B.2)

The eigenvalues of the Jacobian matrix in [Eq. (B.2)](#eq21) are given by the characteristic equation below,

$\left( \lambda+\upsilon_{1} \right)\left( \lambda+\gamma_{1} \right)\left( \lambda+\varepsilon+\upsilon_{1} \right)\left( \lambda^{2}+p\lambda+q \right)=0,$ (B.3)

where, $p=\left( \omega+\delta_{u}+\upsilon_{1} \right)+(\gamma_{1}+\alpha_{1})$ and $q=\left( \omega+\delta_{u}+\upsilon_{1} \right)(\gamma_{1}+\alpha_{1})(1-R_{0h})$_._

It can be seen from [Eq. (B.3)](#eq22) that ${-\upsilon}_{1}$, $-\gamma_{1}$, and $-\left( \varepsilon+\upsilon_{1} \right)$ are negative eigenvalues of $J\left( E_{0h} \right)$, while the other two eigenvalues are defined by the quadratic equation and are negative provided $R_{0h}<1$. The SH sub-model [Eq. (B.1)](#eqB1) is locally asymptotically stable for $R_{0h}<1$ because all of its eigenvalues at $E_{0h}$ are negative.

**1.2 Existence of endemic equilibrium for the SH sub-model**

*Lemma 1. The SH sub-model* [*Eq. (B.1)*](#eqB1) *possesses a unique endemic equilibrium* $(E_{1h})$ *if* $R_{0h}>1$.

Lemma 1 can be supported by the understanding that the SH sub-model system attains a distinct endemic equilibrium ($E_{1h}$) only when $R_{0h}>1$. This condition implies that the potential for sustained transmission of *Schistosoma haematobium* infections within the population is determined by whether the reproductive rate of the parasite surpasses its control factors. Therefore, when $R_{0h}>1$, $E_{1h}$ emerges as the dominant outcome, signifying the potential establishment and persistence of *Schistosoma* *haematobium* infections in the population.

**1.3 Global stability of the SH sub-model**

The global stability for the SH sub-model can be computed using the center manifold theory [[48](#rf48)] to determine the local asymptotic stability or nature of the bifurcation of the endemic equilibrium.

Theorem 3. *The endemic equilibrium* $E_{1h}$ *of SH sub-model* [*Eq. (B.1)*](#eqB1) *is locally asymptotically stable if* $R_{0}>1$ *and the bifurcation parameter* $\beta_{u}=\beta_{u}^{*}>\frac{\upsilon_{1}\gamma_{1}(\gamma_{1}+\alpha_{1})\left( \omega+\delta_{u}+\upsilon_{1} \right)}{{\beta_{1}\Lambda_{1}\Lambda}_{h}}$.

**Proof**: We transform the SH sub-model [Eq. (B.1)](#eqB1), where $H=x_{1}$, $I_{h}=x_{2}$, $R_{h}=x_{3}$, $S_{1}=x_{4}$, and $I_{1}=x_{5}$ with the vector notation $X={(x_{i})}^{T}, i=1,2,3,4,5$ and $\frac{dX}{\mathrm{dt}}=\boldsymbol{F}=:$ ${(f_{1}, f_{2}, f_{3}, f_{4}, f_{5} )}^{T}$. Thus, the following model system is obtained

| $x_{1}^{'}=:f_{1}=\Lambda_{h}+\varepsilon x_{3}-\beta_{u}x_{1}x_{5}-\upsilon_{1}x_{1}$ |  |
| --- | --- |
| $x_{2}^{'}=:f_{2}=\beta_{u}x_{1}x_{5}-\left( \omega+\delta_{u}+\upsilon_{1} \right)x_{2}$ |  |
| $x_{3}^{'}=:f_{3}=\omega x_{2}-\left( \varepsilon+\upsilon_{1} \right)x_{3}$ | (B.4) |
| $x_{4}^{'}=:f_{4}=\Lambda_{1}-\beta_{1}x_{2}x_{4}-\gamma_{1}x_{4}$ |  |
| $x_{5}^{'}=:f_{5}=\beta_{1}x_{2}x_{4}-(\gamma_{1}+\alpha_{1})x_{5}$ |  |

The linearized SH sub-model [Eq. (B.4)](#eq23) evaluated at disease-free equilibrium $E_{0h}=\left( x_{1}=\frac{\Lambda_{h}}{\upsilon_{1}}, x_{2}=0, x_{3}=0, x_{4}=\frac{S_{1}}{\gamma_{1}}, x_{5}=0 \right)$ with the bifurcation parameter $\beta_{u}=\beta_{u}^{*}=\frac{\upsilon_{1}\gamma_{1}(\gamma_{1}+\alpha_{1})\left( \omega+\delta_{u}+\upsilon_{1} \right)}{{\beta_{1}\Lambda_{1}\Lambda}_{h}}$, has a simple eigenvalue associated with a right eigenvector $\boldsymbol{u=}{\boldsymbol{(}u_{1}\boldsymbol{,}u_{2}\boldsymbol{,}u_{3}\boldsymbol{,}u_{4}\boldsymbol{,}u_{5}\boldsymbol{)}}^{T}$and a left eigenvector $\boldsymbol{w=(}w_{1}\boldsymbol{,}w_{2}\boldsymbol{,}w_{3}\boldsymbol{,}w_{4}\boldsymbol{,}w_{5}\boldsymbol{)}$ satisfying $\boldsymbol{u.w=1}$, where

$$\boldsymbol{u=}\left( \begin{matrix} u_{1}=\frac{\varepsilon\omega\upsilon_{1}\gamma_{1}\left( \gamma_{1}+\alpha_{1} \right)-{\beta_{1}\beta_{u}\Lambda_{1}\Lambda}_{h}\left( \varepsilon+\upsilon_{1} \right)}{\gamma_{1}\left( \gamma_{1}+\alpha_{1} \right)\left( \varepsilon+\upsilon_{1} \right)u_{1}^{2}}u_{2}, & u_{2}>0, & u_{3}=\frac{\omega}{\left( \varepsilon+\upsilon_{1} \right)}u_{2}, & u_{4}=\frac{-\beta_{1}\Lambda_{1}}{\gamma_{1}^{2}}u_{2}, & u_{5}= \end{matrix}\frac{\beta_{1}\Lambda_{1}}{\gamma_{1}\left( \gamma_{1}+\alpha_{1} \right)}u_{2} \right)^{\boldsymbol{T}}$$

$\boldsymbol{w=}\left( \begin{matrix} w_{1}=0 & w_{2}>0 & w_{3}=0 & w_{4}=0 & w_{5}= \end{matrix}\frac{\beta_{u}\Lambda_{h}}{\upsilon_{1}(\gamma_{1}+\alpha_{1})}w_{2} \right)$

Thus, based on $\boldsymbol{w}$, it can be seen that the second-order partial derivatives, $f_{k}$ for$k=1, 3, 4$ associated with left eigenvalues will varnish at the disease-free equilibrium, as a result,

$\begin{matrix} a=w_{2}u_{1}u_{5}\frac{\partial^{2}f_{2}\left( 0,0 \right)}{\partial x_{1}\partial x_{5}}+w_{5}u_{2}u_{4}\frac{\partial^{2}f_{5}\left( 0,0 \right)}{\partial x_{2}\partial x_{4}}=\left( \frac{\omega\varepsilon\gamma_{1}\beta_{1}\left( \gamma_{1}+\alpha_{1} \right)-{\beta_{u}\Lambda}_{h}\left( \varepsilon+\upsilon_{1} \right)\left( \gamma_{1}\upsilon_{1}+\alpha_{1}\upsilon_{1}+\Lambda_{1}\beta_{1}^{2} \right)}{\left( \varepsilon+\upsilon_{1} \right)\left( \gamma_{1}+\alpha_{1} \right)^{2}\upsilon_{1}^{2}\gamma_{1}^{2}} \right)\beta_{1}\Lambda_{1}w_{2}u_{2}^{2} \\ b=w_{2}u_{1}\frac{\partial^{2}f_{2}(0,0)}{\partial x_{1}\partial\beta_{u}^{*}}+w_{2}u_{5}\frac{\partial^{2}f_{2}(0,0)}{\partial x_{5}\partial\beta_{u}^{*}}=\frac{\beta_{1}\Lambda_{1}\Lambda_{h}}{\gamma_{1}\upsilon_{1}\left( \gamma_{1}+\alpha_{1} \right)}w_{2}u_{2} \end{matrix}$

It is evident that $b>0$, and the local dynamics around the disease-free equilibrium point $E_{0h}$ for $\beta_{u}=\beta_{u}^{*}$ depend on the sign of the coefficient $a$. Consequently, if $\omega\varepsilon\gamma_{1}\beta_{1}\left( \gamma_{1}+\alpha_{1} \right)>{\beta_{u}\Lambda}_{h}\left( \varepsilon+\upsilon_{1} \right)\left( \gamma_{1}\upsilon_{1}+\alpha_{1}\upsilon_{1}+\Lambda_{1}\beta_{1}^{2} \right)$, a backward bifurcation occurs. This leads to the absence of global stability for the disease-free equilibrium and the presence of an endemic equilibrium. Conversely, if the condition $\omega\varepsilon\gamma_{1}\beta_{1}\left( \gamma_{1}+\alpha_{1} \right)<{\beta_{u}\Lambda}_{h}\left( \varepsilon+\upsilon_{1} \right)\left( \gamma_{1}\upsilon_{1}+\alpha_{1}\upsilon_{1}+\Lambda_{1}\beta_{1}^{2} \right)$ holds, a forward bifurcation arises at $R_{0h}=1$, and the disease-free equilibrium becomes globally stable. In this scenario, the disease-free equilibrium is the only local attractor, and an endemic equilibrium does not occur. Furthermore, analyzing the terms $\omega\varepsilon\gamma_{1}\beta_{1}\left( \gamma_{1}+\alpha_{1} \right)$ and ${\beta_{u}\Lambda}_{h}\left( \varepsilon+\upsilon_{1} \right)\left( \gamma_{1}\upsilon_{1}+\alpha_{1}\upsilon_{1}+\Lambda_{1}\beta_{1}^{2} \right)$ reveals the significance of individual parameters in influencing the occurrence of forward or backward bifurcations.

1. **SM sub model**

The following equations represent the SM sub-model

| $H^{'}=\Lambda_{h}+\alpha R_{m}-\beta_{i}I_{2}H-\upsilon_{1}H$ |  |
| --- | --- |
| $I_{m}^{'}=\beta_{i}I_{2}H-(\gamma+\delta_{i}+\upsilon_{1})I_{m}$ |  |
| $R_{m}^{'}=\gamma I_{m}-\left( \alpha+\upsilon_{1} \right)R_{m}$ | (B.5) |
| $S_{2}^{'}=\Lambda_{2}-\beta_{2}I_{m}S_{2}-\gamma_{2}S_{2}$ |  |
| $I_{2}^{'}=\beta_{2}I_{m}S_{2}-(\gamma_{2}+\alpha_{2})I_{2}$ |  |

The same procedures used to examine the SH sub-model in [Eq. (B.1)](#eqB1) were also used to analyze the SM sub-model in [Eq. (B.5)](#eq24).

**2.1 Local stability of the SM sub-model**

Theorem 4. *The SM sub-model* [*Eq. (B.5)*](#eq24)*, at* $E_{0m}$*, is locally asymptotically stable if* $R_{0m}<1$*, otherwise unstable for* $R_{0m}>1$*.*

**Proof**: The SH sub-model has the following Jacobian matrix at $E_{0m}$

$J\left( E_{0m} \right)=\left( \begin{matrix} -\upsilon_{1} & 0 & \alpha& 0 & \frac{{-\beta_{i}\Lambda}_{h}}{\upsilon_{1}} \\ 0 & -(\gamma+\delta_{i}+\upsilon_{1}) & 0 & 0 & \frac{{\beta_{i}\Lambda}_{h}}{\upsilon_{1}} \\ 0 & \gamma& -\left( \alpha+\upsilon_{1} \right) & 0 & 0 \\ 0 & \frac{{-\beta_{2}\Lambda}_{2}}{\gamma_{2}} & 0 & -\gamma_{2} & 0 \\ 0 & \frac{{\beta_{2}\Lambda}_{2}}{\gamma_{2}} & 0 & 0 & -(\gamma_{2}+\alpha_{2}) \end{matrix} \right)$ (B.6)

Clearly, in Jacobian matrix [Eq. (B.6)](#eq25), ${-\upsilon}_{1}$, $-\gamma_{2}$, and $-\left( \alpha+\upsilon_{1} \right)$ are negative eigenvalues of $J\left( E_{0m} \right)$. The resultant quadratic equation $\left( \lambda^{2}+\left[ (\gamma+\delta_{i}+\upsilon_{1})+(\gamma_{2}+\alpha_{2}) \right]\lambda+(\gamma+\delta_{i}+\upsilon_{1})(\gamma_{2}+\alpha_{2})(1-R_{0m}) \right)=0$, has two negative eigenvalues provided $R_{0h}<1$. Thus, SM sub-model [Eq. (B.5)](#eq24) is locally stable as long as all eigenvalues at $E_{0m}$ are negative

**2.2 Existence of endemic equilibrium for the SM sub-model**

Lemma 2*. If* $R_{0m}>1$*, the SM sub-model* [*Eq. (B.5)*](#eq24) *has an endemic equilibrium (*$E_{1m}$*).*

The computation of the endemic equilibrium point $E_{1m}=(H^{'*}$,$I_{m}^{'*}$, $R_{m}^{'*}$, $S_{2}^{'*}; I_{2}^{'*})$ results,

$H^{'*}=\frac{(\gamma_{2}+\alpha_{2})\left( (\alpha+\upsilon_{1})\Lambda_{2}+\alpha\gamma I_{m} \right)(\beta_{2}I_{m}+\gamma_{2})}{(\alpha+\upsilon_{1})(\gamma_{2}+\alpha_{2})(\beta_{2}I_{m}+\gamma_{2})}$, $R_{m}^{'*}=\frac{\omega}{(\alpha+\upsilon_{1})}I_{m}$, $S_{2}^{'*}=\frac{\Lambda_{2}}{(\beta_{2}I_{m}+\gamma_{2})}I_{m}$, $I_{2}^{'*}=\frac{\beta_{2}\Lambda_{2}I_{m}}{(\gamma_{2}+\alpha_{2})(\beta_{2}I_{m}+\gamma_{2})}I_{m}$

The endemic equilibrium of the SM sub-model [Eq. (B.5)](#eq24) is satisfied by the resultant polynomial provided in equation [Eq. (B.7)](#eq26),

$\lambda^{3}+m_{2}\lambda^{2}+m_{1}\lambda+m_{0}=0$ (B.7)

where $m_{0}=-\frac{\gamma_{2}\beta_{i}\beta_{2}\Lambda_{2}\Lambda_{h}}{\beta_{2}(\gamma_{2}+\alpha_{2})(\gamma+\delta_{i}+\upsilon_{1})}$, $m_{1}=\frac{(\gamma_{2}+\alpha_{2})(\gamma+\delta_{i}+\upsilon_{1})\left( \gamma_{2}-\beta_{2}\Lambda_{h} \right)-\gamma_{2}\beta_{i}\beta_{2}\Lambda_{2}}{\beta_{2}(\gamma_{2}+\alpha_{2})(\gamma+\delta_{i}+\upsilon_{1})}$, $m_{2}=\frac{(\gamma_{2}+\alpha_{2})(\gamma+\delta_{i}+\upsilon_{1})\left( \gamma_{2}-\beta_{2}\Lambda_{h} \right)-(\gamma_{2}+\alpha_{2})\alpha\gamma\gamma_{2}\beta_{i}\beta_{2}\Lambda_{2}}{\beta_{2}\gamma_{2}(\gamma_{2}+\alpha_{2})(\gamma+\delta_{i}+\upsilon_{1})}$. There is no doubt that $m_{0}<0$, and in line with Descartes' rule of signs [[3](#srf3)], if either or both of $m_{i}>0,i=1,2$ results in at least one positive root for equation [Eq. (B.7)](#eq26), and thus endemic equilibrium occurs.

**2.3 Global stability of the SM sub-model**

Theorem 5. *The endemic equilibrium* $E_{1m}$ *of SM sub-model* [*Eq. (B.5)*](#eq24) *is locally asymptotically stable if* $R_{0}>1$ *and the bifurcation parameter* $\beta_{i}=\beta_{i}^{*}>\frac{\upsilon_{1}\gamma_{2}(\gamma_{2}+\alpha_{2})(\gamma+\delta_{i}+\upsilon_{1})}{{\beta_{2}\Lambda_{1}\Lambda}_{h}}$.

**Proof**: We transform the SM sub-model [Eq. (B.5)](#eq24) according to Castillo and Songs [[4](#srf4)], where $H=y_{1}$, $I_{m}=y_{2}$, $R_{m}=y_{3}$, $S_{2}=y_{4}$, and $I_{2}=y_{5}$ with the vector notation $\boldsymbol{Y}={(y_{i})}^{T}, i=1,2,3,4,5$ and $\frac{dy}{\mathrm{dt}}=\boldsymbol{G}=:$ ${(g_{1}, g_{2}, g_{3}, g_{4}, g_{5} )}^{T}$. Thus, the new SM sub-model system Eq. () is obtained

| $y_{1}^{'}=:g_{1}=\Lambda_{h}+\alpha y_{3}-\beta_{i}y_{1}y_{5}-\upsilon_{1}y_{1}$ |  |
| --- | --- |
| $y_{2}^{'}=:g_{2}=\beta_{i}y_{1}y_{5}-(\gamma+\delta_{i}+\upsilon_{1})y_{2}$ |  |
| $y_{3}^{'}=:g_{3}=\gamma y_{2}-\left( \alpha+\upsilon_{1} \right)y_{3}$ | (B.8) |
| $y_{4}^{'}=:g_{4}=\Lambda_{2}-\beta_{2}y_{2}y_{4}-\gamma_{2}y_{4}$ |  |
| $y_{5}^{'}=:g_{5}=\beta_{2}y_{2}y_{4}-(\gamma_{2}+\alpha_{2})y_{5}$ |  |

The linearized Jacobian matrix of the SM sub-model in [Eq. (B.8)](#eq27) at $E_{0m}$ and $\beta_{i}=\beta_{i}^{*}=\frac{\upsilon_{1}\gamma_{2}(\gamma_{2}+\alpha_{2})(\gamma+\delta_{i}+\upsilon_{1})}{{\beta_{2}\Lambda_{2}\Lambda}_{h}}$ has a simple eigenvalue associated with a right ($\boldsymbol{v}$**)** and a left ($\boldsymbol{\eta}$**)** eigenvector satisfying $\boldsymbol{v.\eta=1}$, where

$\boldsymbol{v}\boldsymbol{=}\left( \begin{matrix} v_{1}=\frac{\alpha\upsilon_{1}\gamma\gamma_{2}\left( \gamma_{2}+\alpha_{2} \right)-{\beta_{2}\beta_{i}\Lambda_{2}\Lambda}_{h}\left( \alpha+\upsilon_{1} \right)}{\gamma_{2}\left( \gamma_{2}+\alpha_{2} \right)\left( \alpha+\upsilon_{1} \right)u_{1}^{2}}v_{2}, & v_{2}>0, & v_{3}=\frac{\alpha}{\left( \alpha+\upsilon_{1} \right)}v_{2}, & v_{4}=\frac{-\beta_{2}\Lambda_{2}}{\gamma_{2}^{2}}v_{2}, & v_{5}= \end{matrix}\frac{\beta_{2}\Lambda_{2}}{\gamma_{2}\left( \gamma_{2}+\alpha_{2} \right)}v_{2} \right)^{\boldsymbol{T}}$

$\boldsymbol{\eta}\boldsymbol{=}\left( \begin{matrix} \eta_{1}=0 & \eta_{2}>0 & \eta_{3}=0 & \eta_{4}=0 & \eta_{5}= \end{matrix}\frac{\beta_{i}\Lambda_{h}}{\upsilon_{1}(\gamma_{2}+\alpha_{2})}\eta_{2} \right)$

The coefficients $a$ and $b$ as defined in Castillo and Songs [[4](#srf4)], are given as

$\begin{matrix} a=\eta_{2}v_{1}v_{5}\frac{\partial^{2}f_{2}\left( 0,0 \right)}{\partial x_{1}\partial x_{5}}+\eta_{5}v_{2}v_{4}\frac{\partial^{2}f_{5}\left( 0,0 \right)}{\partial x_{2}\partial x_{4}}=\left( \frac{\alpha\upsilon_{1}\gamma_{2}\beta_{2}\left( \gamma_{1}+\alpha_{1} \right)-{\beta_{i}\Lambda}_{h}\left( \alpha+\upsilon_{1} \right)\left( \gamma_{2}\upsilon_{1}+\alpha_{2}\upsilon_{1}+\Lambda_{2}\beta_{2}^{2} \right)}{\left( \alpha+\upsilon_{1} \right)\left( \gamma_{2}+\alpha_{2} \right)^{2}\upsilon_{1}^{2}\gamma_{2}^{2}} \right)\beta_{2}\Lambda_{2}\eta_{2}v_{2}^{2} \\ b=\eta_{2}v_{1}\frac{\partial^{2}f_{2}(0,0)}{\partial x_{1}\partial\beta_{u}^{*}}+\eta_{2}v_{5}\frac{\partial^{2}f_{2}(0,0)}{\partial x_{5}\partial\beta_{u}^{*}}=\frac{\beta_{2}\Lambda_{2}\Lambda_{h}}{\gamma_{2}\upsilon_{1}\left( \gamma_{2}+\alpha_{2} \right)}\eta_{2}v_{2} \end{matrix}$

Consequently, $b$ is positive, and the local stability dynamics are determined by the sign of coefficient $a$. Thus, backward bifurcation occurs if and only if $a>0$, given that $\alpha\upsilon_{1}\gamma_{2}\beta_{2}\left( \gamma_{1}+\alpha_{1} \right)>{\beta_{i}\Lambda}_{h}\left( \alpha+\upsilon_{1} \right)\left( \gamma_{2}\upsilon_{1}+\alpha_{2}\upsilon_{1}+\Lambda_{2}\beta_{2}^{2} \right)$, On the other hand, a forward bifurcation takes place whenever $a<0$.

1. **Co-dynamics model system**

**3.1 Local stability of the co-dynamics model**

Theorem 6. *The SHM co-infection model* [*Eq. (1-11*](#eq111)*)* *at* $E_{0hm}$*, is locally asymptotically stable if* $R_{0hm}<1$*, otherwise unstable for* $R_{0hm}>1$

**Proof:** We evaluate the Jacobian matrix $J(E_{0hm})$ at $E_{0hm}$ given in equation [Eq. (B.9)](#eq28)

$\left( \begin{matrix} \boldsymbol{-}\upsilon_{1} & \boldsymbol{0} & \boldsymbol{0} & \boldsymbol{0} & \alpha& \varepsilon& \theta& \boldsymbol{0} & \frac{{-\beta_{u}\Lambda}_{h}}{\upsilon_{1}} & \boldsymbol{0} & \frac{{-\beta_{i}\Lambda}_{h}}{\upsilon_{1}} \\ \boldsymbol{0} & \boldsymbol{-}\rho_{1} & \boldsymbol{0} & \boldsymbol{0} & \boldsymbol{0} & \boldsymbol{0} & \boldsymbol{0} & \boldsymbol{0} & \boldsymbol{0} & \boldsymbol{0} & \frac{{\beta_{i}\Lambda}_{h}}{\upsilon_{1}} \\ \boldsymbol{0} & \boldsymbol{0} & \boldsymbol{-}\rho_{2} & \boldsymbol{0} & \boldsymbol{0} & \boldsymbol{0} & \boldsymbol{0} & \boldsymbol{0} & \frac{{\beta_{u}\Lambda}_{h}}{\upsilon_{1}} & \boldsymbol{0} & \boldsymbol{0} \\ \boldsymbol{0} & \boldsymbol{0} & \boldsymbol{0} & -\rho_{3} & \boldsymbol{0} & \boldsymbol{0} & \boldsymbol{0} & \boldsymbol{0} & \boldsymbol{0} & \boldsymbol{0} & \boldsymbol{0} \\ \boldsymbol{0} & \gamma& \boldsymbol{0} & \tau_{1}\left( 1-\delta\right) & -\left( \alpha+\upsilon_{1} \right) & \boldsymbol{0} & \boldsymbol{0} & \boldsymbol{0} & \boldsymbol{0} & \boldsymbol{0} & \boldsymbol{0} \\ \boldsymbol{0} & \boldsymbol{0} & \omega& \rho_{4} & \boldsymbol{0} & -\left( \varepsilon+\upsilon_{1} \right) & \boldsymbol{0} & \boldsymbol{0} & \boldsymbol{0} & \boldsymbol{0} & \boldsymbol{0} \\ \boldsymbol{0} & \boldsymbol{0} & \boldsymbol{0} & \delta& \boldsymbol{0} & \boldsymbol{0} & -\left( \theta+\upsilon_{1} \right) & \boldsymbol{0} & \boldsymbol{0} & \boldsymbol{0} & \boldsymbol{0} \\ \boldsymbol{0} & \boldsymbol{0} & \frac{{-\beta_{1}\Lambda}_{1}}{\gamma_{1}} & \frac{{-\beta_{1}\Lambda}_{1}}{\gamma_{1}} & \boldsymbol{0} & \boldsymbol{0} & \boldsymbol{0} & -\gamma_{1} & \boldsymbol{0} & \boldsymbol{0} & \boldsymbol{0} \\ \boldsymbol{0} & \boldsymbol{0} & \frac{{\beta_{1}\Lambda}_{1}}{\gamma_{1}} & \frac{{\beta_{1}\Lambda}_{1}}{\gamma_{1}} & \boldsymbol{0} & \boldsymbol{0} & \boldsymbol{0} & \boldsymbol{0} & -(\gamma_{1}+\alpha_{1}) & \boldsymbol{0} & \boldsymbol{0} \\ \boldsymbol{0} & \frac{{-\beta_{2}\Lambda}_{1}}{\gamma_{2}} & \boldsymbol{0} & \frac{{-\beta_{2}\Lambda}_{1}}{\gamma_{2}} & \boldsymbol{0} & \boldsymbol{0} & \boldsymbol{0} & \boldsymbol{0} & \boldsymbol{0} & -\gamma_{2} & \boldsymbol{0} \\ \boldsymbol{0} & \frac{{\beta_{2}\Lambda}_{1}}{\gamma_{2}} & \boldsymbol{0} & \frac{{\beta_{2}\Lambda}_{1}}{\gamma_{2}} & \boldsymbol{0} & \boldsymbol{0} & \boldsymbol{0} & \boldsymbol{0} & \boldsymbol{0} & \boldsymbol{0} & -(\gamma_{2}+\alpha_{2}) \end{matrix} \right)$ (B.9)

where $\rho_{1}=(\gamma+\delta_{i}+\upsilon_{1})$, $\rho_{2}=\left( \omega+\delta_{u}+\upsilon_{1} \right)$, $\rho_{3}=(\delta+\delta_{i}+\delta_{u}+\upsilon_{1})$, $\rho_{4}={(1-\tau}_{1})\left( 1-\delta\right)$. The eigenvalues of the SHM co-infection model system that have negative real parts are $\boldsymbol{-}\upsilon_{1}$, $-\left( \omega+\delta_{u}+\upsilon_{1} \right)$, $-\left( \alpha+\upsilon_{1} \right)$, $-\left( \varepsilon+\upsilon_{1} \right)$, $-\left( \theta+\upsilon_{1} \right)$, $-\gamma_{1}$, $-(\gamma_{1}+\alpha_{1})$, and $-\gamma_{2}$. The Jacobian in [Eq. (B.9)](#eq28) can be solved to obtain the remaining three eigenvalues, given by the polynomial in equation [Eq. (B.10)](#eq29).

$\lambda^{3}+a_{2}\lambda^{2}+a_{1}\lambda+a_{0}=0$ (B.10)

where $a_{0}=\left( \gamma+\delta_{i}+\upsilon_{1} \right)\left( \delta+\delta_{i}+\delta_{u}+\upsilon_{1} \right)\left( \gamma_{2}+\alpha_{2} \right)-\beta_{i}\beta_{2}\left( \delta+\delta_{i}+\delta_{u}+\upsilon_{1} \right){\Lambda_{1}\Lambda}_{h}$

$$a_{1}=\left( \gamma+\delta_{i}+\upsilon_{1} \right)\left( \delta+\delta_{i}+\delta_{u}+\upsilon_{1} \right)+\left( \delta+\delta_{i}+\delta_{u}+\upsilon_{1} \right)\left( \gamma_{2}+\alpha_{2} \right)+\left( \gamma_{2}+\alpha_{2} \right)\left( \gamma+\delta_{i}+\upsilon_{1} \right)-\beta_{i}\beta_{2}{\Lambda_{1}\Lambda}_{h}$$

$$a_{2}=\left( \gamma+\delta_{i}+\upsilon_{1} \right)+\left( \delta+\delta_{i}+\delta_{u}+\upsilon_{1} \right)+\left( \gamma_{2}+\alpha_{2} \right)$$

Routh-Hurwitz stability criterion states that equatioin [Eq. (B.10)](#eq29) has negative roots only when $a_{0}>0$, $a_{1}>0$, $a_{2}>0$, and $a_{2}a_{1}>a_{0}$. Thus, the *S.haematobium-mansoni* co-infection model is locally asymptotically stable.

**3.2 Global stability of the co-infection model**

To determine whether endemic equilibrium is asymptotic at the local level, we use the centre manifold theory in Castillo and Song [[4](#srf4)]. Let $H=z_{1}$, $I_{m}=z_{2}$,$I_{h}=z_{3}$,$I_{hm}=z_{4}$, $R_{m}=z_{5}$,$R_{h}=z_{6}$, $R_{hm}=z_{7}$, $S_{1}=z_{8}$, $I_{1}=z_{9}$, $S_{2}=z_{10}$, and $I_{2}=z_{11}$ and with the vector notation $Z={(z_{i})}^{T}, i=1,2,3,4,5$ and $\frac{dZ}{\mathrm{dt}}=\boldsymbol{\psi}=:$ ${(\psi_{i} )}^{T}, i=1,$2, …,11. Thus, the resultant transformed *S. haematobium-mansoni* co-infection in model (*Eq25*) exhibits the same disease-free equilibrium point ($E_{0hm}$) as the original sytem in equations [Eq. (1-11](#eq111)).

| $z_{1}^{'}=\psi_{1}:=\Lambda_{h}+\varepsilon z_{6}+\alpha z_{5}+\theta z_{7}-(\beta_{i}z_{11}+\beta_{u}z_{9})z_{1}-\upsilon_{1}z_{1}$ |  |
| --- | --- |
| $z_{2}^{'}=\psi_{2}:=\beta_{i}z_{11}z_{1}-\beta_{u}z_{9}z_{2}-(\gamma+\delta_{i}+\upsilon_{1})z_{2}$ |  |
| $z_{3}^{'}=\psi_{3}:=\beta_{u}z_{9}z_{1}-\beta_{i}z_{11}z_{3}-\left( \omega+\delta_{u}+\upsilon_{1} \right)z_{3}$ |  |
| $z_{4}^{'}=\psi_{4}:=\beta_{u}z_{9}z_{2}+\beta_{i}z_{11}z_{3}-(\delta+\delta_{i}+\delta_{u}+\upsilon_{1})z_{4}$ |  |
| $z_{5}^{'}=\psi_{5}:=\gamma z_{2}+\tau_{1}\left( 1-\delta\right)z_{4}-\left( \alpha+\upsilon_{1} \right)z_{5}$ | (B.11) |
| $z_{6}^{'}=\psi_{6}:=\omega z_{3}+{(1-\tau}_{1})\left( 1-\delta\right)z_{4}-\left( \varepsilon+\upsilon_{1} \right)z_{6}$ |  |
| $z_{7}^{'}=\psi_{7}:=\delta z_{4}-\left( \theta+\upsilon_{1} \right)z_{7}$ |  |
| $z_{8}^{'}=\psi_{8}:=\Lambda_{1}-\beta_{1}(z_{3}+z_{4})z_{8}-\gamma_{1}z_{8}$ |  |
| $z_{9}^{'}=\psi_{9}:=\beta_{1}(z_{3}+z_{4})z_{8}-(\gamma_{1}+\alpha_{1})z_{9}$ |  |
| $z_{10}^{'}=\psi_{10}:=\Lambda_{2}-\beta_{2}(z_{2}+z_{4})z_{10}-\gamma_{2}z_{10}$ |  |
| $z_{11}^{'}=\psi_{11}:=\beta_{2}(z_{2}+z_{4})z_{10}-(\gamma_{2}+\alpha_{2})z_{11}$ |  |

Therefore, in the linearized Jacobian matrix of the SHM sub-model, [Eq. (B.8)](#eq27) at $E_{0hm}$ and $\beta_{i}=\beta_{i}^{*}$ as a bifurcation parameter, a straightforward eigenvalue is associated with both the right eigenvector ($\boldsymbol{u}$**)** and the left eigenvector ($\boldsymbol{v}$**)**, with the condition $\boldsymbol{u.v=1}$. Thus eigenvectors $\boldsymbol{u}$ and $\boldsymbol{v}$ are given as

$\boldsymbol{u=}\left( \begin{matrix} u_{1} \\ u_{2} \\ u_{3} \\ u_{4} \\ u_{5} \\ u_{6} \\ u_{7} \\ u_{8} \\ u_{9} \\ u_{10} \\ u_{11} \end{matrix} \right)=\left( \begin{matrix} r_{1}u_{2}+r_{2}u_{3} \\ u_{2}>0 \\ u_{3}>0 \\ 0 \\ \left( \frac{\gamma}{\alpha+\upsilon_{1}} \right)u_{2} \\ \left( \frac{\omega}{\alpha+\upsilon_{1}} \right)u_{3} \\ 0 \\ \left( \frac{-\beta_{u}{\beta_{1}\Lambda}_{1}}{\gamma_{1}} \right)u_{3} \\ \left( \frac{\beta_{u}{\beta_{1}\Lambda}_{1}}{\gamma_{1}+\alpha_{1}} \right)u_{3} \\ \left( \frac{\beta_{i}{\beta_{2}\Lambda}_{1}}{\gamma_{2}} \right)u_{2} \\ \left( \frac{\beta_{u}{\beta_{1}\Lambda}_{1}}{(\gamma_{2}+\alpha_{2})} \right)u_{2} \end{matrix} \right)$, $\boldsymbol{v=}\left( \begin{matrix} v_{1} \\ v_{2} \\ v_{3} \\ v_{4} \\ v_{5} \\ v_{6} \\ v_{7} \\ v_{8} \\ v_{9} \\ v_{10} \\ v_{11} \end{matrix} \right)=\left( \begin{matrix} 0 \\ v_{2}>0 \\ v_{3}>0 \\ r_{3}v_{2} \\ 0 \\ 0 \\ 0 \\ 0 \\ \left( \frac{\beta_{i}\Lambda_{h}}{\gamma_{2}} \right)v_{3} \\ 0 \\ \left( \frac{\beta_{u}\Lambda_{h}}{\gamma_{1}+\alpha_{1}} \right)v_{3} \end{matrix} \right)$

where $r_{1}=\left( \frac{\alpha\gamma}{\alpha+\upsilon_{1}}-\frac{{\beta_{i}{\beta_{2}\Lambda}_{1}\Lambda}_{h}}{(\gamma_{2}+\alpha_{2})} \right)$, $r_{2}=\left( \frac{\varepsilon\omega}{\varepsilon+\upsilon_{1}}-\frac{{\beta_{u}{\beta_{1}\Lambda}_{1}\Lambda}_{h}}{(\gamma_{1}+\alpha_{1})} \right)$ and $r_{3}=\frac{(\gamma_{2}+\alpha_{2})(\beta_{u}{\beta_{1}\Lambda}_{1}\Lambda_{h}v_{3}+(\gamma_{1}+\alpha_{1})\beta_{i}{\beta_{2}\Lambda}_{1}\Lambda_{h})v_{2}}{(\gamma_{1}+\alpha_{1})(\gamma_{2}+\alpha_{2})(\delta+\delta_{i}+\delta_{u}+\upsilon_{1})}$.

Hence, upon examining $\boldsymbol{v}$, it becomes evident that the second-order partial derivatives, denoted as $\psi_{k}$ for$k=1, 5, 6,7,8,10$, linked to the left eigenvalues, will vanish. Consequently, only the partial derivatives associated with $k=2, 3, 4, 9$ and $11$ at the disease-free equilibrium are taken into consideration. This leads to the determination of the coefficients $a$ and $b$ as follows:

$\begin{matrix} a=v_{2}u_{2}u_{9}\frac{\partial^{2}\psi_{2}\left( 0,0 \right)}{\partial z_{2}\partial z_{9}}+v_{2}u_{3}u_{11}\frac{\partial^{2}\psi_{2}\left( 0,0 \right)}{\partial z_{3}\partial z_{11}}+v_{3}u_{3}u_{11}\frac{\partial^{2}\psi_{3}\left( 0,0 \right)}{\partial z_{3}\partial z_{11}}+v_{4}u_{2}u_{9}\frac{\partial^{2}\psi_{4}\left( 0,0 \right)}{\partial z_{2}\partial z_{9}}+v_{9}u_{3}u_{8}\frac{\partial^{2}\psi_{9}\left( 0,0 \right)}{\partial z_{3}\partial z_{8}}+v_{11}u_{2}u_{10}\frac{\partial^{2}\psi_{11}\left( 0,0 \right)}{\partial z_{2}\partial z_{10}} \end{matrix}$

$$=\left( \frac{\beta_{u}\beta_{1}\beta_{2}\Lambda_{1}}{\gamma_{2}+\alpha_{2}}+\frac{(r_{3}-1)\beta_{1}\beta_{u}^{2}\Lambda_{1}}{\gamma_{1}+\alpha_{1}} \right)v_{2}u_{2}u_{3}+\frac{\beta_{1}^{2}\beta_{2}^{2}\Lambda_{1}\Lambda_{h}}{\gamma_{2}^{2}}v_{2}u_{2}^{2}+ \frac{\beta_{u}\beta_{2}\Lambda_{1}\left[ \gamma_{1}\gamma_{2}\beta_{2}v_{2}u_{2}^{2}-(\gamma_{2}+\alpha_{2})\beta_{i}\Lambda_{2}v_{3}u_{3}^{2} \right]}{\gamma_{1}\gamma_{2}(\gamma_{1}+\alpha_{1})}$$

$$b=v_{9}u_{3}\frac{\partial^{2}\psi_{9}\left( 0,0 \right)}{\partial z_{3}\partial\beta_{i}^{*}} +v_{9}u_{4}\frac{\partial^{2}\psi_{9}\left( 0,0 \right)}{\partial z_{4}\partial\beta_{i}^{*}}=\frac{\beta_{1}\Lambda_{1}\Lambda_{h}}{\gamma_{1}\gamma_{2}}v_{3}^{2}$$

The computation of the coefficient reveals that despite $b>0$, it is not evident that $a$ is also positive. In such instances, the disease-free equilibrium does not exhibit asymptotic stability, and there is a possibility of an endemic equilibrium if $r_{3}>1$ and $\gamma_{1}\gamma_{2}\beta_{2}v_{2}u_{2}^{2}>(\gamma_{2}+\alpha_{2})\beta_{i}\Lambda_{2}v_{3}u_{3}^{2}$. However, if the aforementioned condition holds true, resulting in a positive value for $a$, the SHM co-infection model experiences a backward bifurcation, given that both $a$ and b are positive.

**References**

1. Mangal TD, Paterson S, A. Fenton. Predicting the impact of long-term temperature changes on the epidemiology and control of schistosomiasis: a mechanistic model. PLoS One. 2008;3(1):1438. <https://doi.org/10.1371/journal.pone.0001438>
2. Kalinda C, Chimbari MJ, Mukaratirwa S. Effect of temperature on the *Bulinus globosus*-*Schistosoma haematobium* system. Infect Dis Poverty. 2017;6(1):1-7. <https://doi.org/10.1186/s40249-017-0260-z>
3. Grabiner DJ. Descartes' rule of signs: Another construction. An Math Mon. 1999; 106(9):854-6. <https://doi.org/10.1080/00029890.1999.12005131>
4. Castillo-Chavez C, Song B. Dynamical models of tuberculosis and their applications. Math Biosci Eng. 2004; 1(2):361-404.
